# Supplementary material for: Network mechanisms and dysfunction within an integrated computational model of progression through mitosis in the human cell cycle
Source: PLoS Comput Biol. 2020 Apr 6;16(4):e1007733. doi: 10.1371/journal.pcbi.1007733 (PMC7162553; doi:10.1371/journal.pcbi.1007733)
Supplement: S1 Appendix — (DOCX) [file pcbi.1007733.s001.docx]

**S1 Appendix: Mitotic proteins and the associated gene names**

Gene names are used instead of protein names in the mitotic biopathway (Figure 1) for the ease of defining relationships, biochemical reactions, and governing ODEs. Italicized texts show both gene and protein names based on information from the UniProt (Universal Protein) Resource. Protein complexes use common names and abbreviations (e.g. MPF, APC/C) or gene names separated by a colon (e.g. MPF is CCNB1:CDK1). Gene names suffixed with P denote the phosphorylated proteins, T denotes the total proteins, and no suffixed denotes the free remaining proteins.

*CCNB1; G2/mitotic-specific cyclin-B1*

CCNB1T; total CCNB1

*CDK1; Cyclin-dependent kinase 1*

CDK1P; phosphorylated CDK1 (Y15)

CDK1T; total CDK1

*CDKN1A; Cyclin-dependent kinase inhibitor 1, p21^CIP1^*

CDKN1AT; total CDKN1A

MPF; CCNB1:CDK1 (maturation promoting factor)

preMPF; CCNB1:CDK1P (phosphorylated MPF)

CDKN1A:MPF; p21^CIP1^ and MPF complex

*CDC25C; dual specificity phosphatase Cdc25C (cell division cycle protein 25 homolog C)*

CDC25CP; phosphorylated CDC25C

CDC25CT; total CDC25C

*WEE1; Wee1-like protein kinase*

WEE1P; phosphorylated WEE1

WEE1T; total WEE1

*LMNA; Lamin-A/C*

LMNAP; phosphorylated LMNA

LMNAT; total LMNA

*PLK1; Polo-like kinase 1*

PLK1P; phosphorylated PLK1

PLK1T; total PLK1

*PPase; PP2A:B55 (Protein phosphatase 2A) and/or CDC14A (cell division cycle 14 homolog A)*

PPaseP; phosphorylated PPase

PPaseT; total PPase

*CDC20; Cell division cycle protein 20 homolog*

CDC20P; phosphorylated CDC20

CDC20T; total CDC20

*CDH1; Fizzy-related protein homolog, hCDH1*

CDH1P; phosphorylated CDH1

CDH1T; total CDH1

*APC/C; Anaphase-promoting complex/cyclosome (multiprotein complex)*

APC/CP; phosphorylated APC/C

APC/CT; total APC/C

APC/CP:CDC20; APC/CP and CDC20 complex

APC/C:CDH1; APC/C and CDH1 complex

APC/CP:CDH1; APC/CP and CDH1 complex

APC/CT:CDH1; APC/CT and CDH1 complex

*PTTG1; Securin*

PTTG1P; phosphorylated PTTG1

PTTG1T; total PTTG1

*ESPL1; Separase*
