# Supplementary material for: Network mechanisms and dysfunction within an integrated computational model of progression through mitosis in the human cell cycle
Source: PLoS Comput Biol. 2020 Apr 6;16(4):e1007733. doi: 10.1371/journal.pcbi.1007733 (PMC7162553; doi:10.1371/journal.pcbi.1007733)
Supplement: S2 Appendix — (DOCX) [file pcbi.1007733.s002.docx]

**S2 Appendix: Biochemical Reactions in the Mitotic Cell Cycle Model**

This Appendix describes 15 major biochemical reactions and other related reactions among 12 mitotic proteins and associated protein complexes represented by 27 biochemical species based on the mitotic biopathway of Figure 1. The corresponding reaction rates are denoted by *k_fn_* and *k_rn_*, where *f* represents forward direction (mitosis promoting), *r* represents reverse direction (mitosis arresting), and *n* denotes reaction number (1, …, 15). The rates characterize either the elementary association and dissociation reactions or the phosphorylation and dephosphorylation reactions in the mitotic cell cycle system. We note here that *K_n_* = *k_rn_*/*k_fn_* is a constant when regulatory signals are not present in a reaction; *K_n_* determines the thermodynamic equilibrium of a reversible reaction and is equal to mass action ratio, defined as the ratio of the product of the reactant concentrations and the product of the product concentrations at equilibrium.

In the mitotic biopathway of Figure 1, the synthesis rate of a protein is denoted by *k_sn_* (usually an unphosphorylated protein from *n* = 1, …, 27) and degradation rate of a protein through a process is denoted by *k_dn.m_* (*n* = 1, …, 27; *m* = 1, 2, and 3 denoting self-degradation, degradation medicated by APC/CP:CDC20, and degradation medicated by APC/CT:CDH1, respectively). The model considers 10 syntheses, 20 self-degradations, and 15 APC/C-dependent (APC/CP:CDC20, APC/CT:CDH1) degradations, as established experimentally. We note here that APC/CT:CDH1 is the sum of APC/C:CDH1 and APC/CP:CDH1. The gene and protein names are as listed in S1 Appendix; suffixed P denotes the phosphorylated protein and suffixed T denotes the total protein. We also note here that the mitotic biopathway (Figure 1) does not distinguish between reactions occurring within the cytosolic and nucleus compartments. The mitotic reactions and associated rate constants are described below:

**(1)**

CDK1 binding to CCNB1 (*k_f1_*) results in the formation of mitotic serine/threonine kinase MPF (CCNB1:CDK1), one of the master regulators of cell cycle reactions. The CCNB family members CCNB2 and CCNB3 are not distinguished from CCNB1 in the model. Disassembly (*k_r1_*) of MPF results in free CDK1 and CCNB1, and is driven by a reduction in the CCNB1 level. In the absence of G2 signals, the steady-state level of total CCNB1 is regulated by constant synthesis (*k_s1_*) with self-degradation (*k_d1.1_, k_d3.1_, k_d4.1_*) and enzymatic degradation mediated by APC/CP:CDC20 (*k_d1.2_, k_d3.2_, k_d4.2_*) and APC/CT:CDH1 (*k_d1.3_, k_d3.3_, k_d4.3_*) resulting in oscillation. Additional mechanisms of CCNB1 regulation occurring in G2/M are not accounted for in the model and will be included in future studies. Since total CDK1 remains approximately constant during the cell cycle phases and exists in much excess than CCNB1, the synthesized CCNB1 is assumed to bind instantly to CDK1, with the association rate (*k_f1_*) much higher than the dissociation rate (*k_r1_*) [1].

**(2)**


MPF transitions between active MPF kinase and inactive preMPF form. The transition is regulated by WEE1-mediated phosphorylation (*k_r2_*) or CDC25CP-mediated dephosphorylation (*k_f2_*) of CDK1 on tyrosine 15 (Y15) [1-4]. Additional sites of modification and mechanisms of regulation are not included in the model. CCNB1 within both MPF and preMPF undergoes self-degradation (*k_d3.1_, k_d4.1_*) and enzymatic degradation mediated by APC/CP:CDC20 (*k_d3.2_, k_d4.2_*) and APC/CT:CDH1 (*k_d3.3_, k_d4.3_*) releasing CDK1.

**(3)**


Phosphorylation (*k_f3_*) of the dual specificity phosphatase CDC25C by MPF (S1) and/or PLK1 (S2) results in active CDC25CP. Dephosphorylation (*k_r3_*) of CDC25CP is mediated by cellular phosphatases (PPases) resulting in inactive CDC25C. The CDC25 family members CDC25A and CDC25B are not distinguished from CDC25C in the model. The steady-state level of total CDC25C is regulated by constant synthesis (*k_s8_*) of CDC25C and self-degradation (*k_d7.1_, k_d8.1_*) and enzymatic degradation mediated by APC/CT:CDH1 (*k_d7.3_, k_d8.3_*) of both CDC25C and CDC25CP resulting in oscillation.

**(4)**


Phosphorylation (*k_f4_*) of the serine/threonine kinase WEE1 by MPF (S1) and/or PLK1 (S2) results in inactive WEE1P, which is dephosphorylated (*k_r4_*) by cellular PPases resulting in inactive WEE1. Phosphorylation promotes degradation (*k_d10.1_*) of WEE1P involving the E3 ligase SCF-βTrCP. To mimic this, we set the WEE1P self-degradation rate (*k_d10.1_*) at an order of magnitude higher than the WEE1 self-degradation rate (*k_d9.1_*), i.e. *k_d10.1_* >> *k_d9.1_*. The steady-state level of total WEE1 is regulated by constant synthesis (*k_s9_*) of WEE1 and self-degradation (*k_d9.1_, k_d10.1_*) of both WEE1 and WEE1P resulting in oscillation.

**(5)**

Upon synthesis (*k_s5_*), multiple (*n*) molecules of CDKN1A (p21^CIP1^) associate (*k_r5_*) with MPF resulting in CDKN1A_n_:MPF complex and loss of oscillation. Dissociation (*k_f5_*) occurs upon reduced CDKN1A level, which is mediated in part by decreased synthesis (*k_s5_*) of CDKN1A and increased self-degradation of CDKN1A (*k_d6.1_*) and increased enzymatic degradation of CDKN1A*_n_*:MPF mediated by APC/CP:CDC20 (*k_d6.2_*) restoring MPF-mediated oscillation. The kinetics of inhibition of MPF activity by CDKN1A indicates a 1 to 3 ratio [5], as described in the Results section. The analysis of the kinetic data on MPF and CDKN1A binding provides the estimate of the binding constant *K_5_* = *k_r5_*/*k_f5_* which provides the thermodynamic constraint for the rate constants *k_r5_* and *k_f5_*.

**(6)**


Phosphorylation (*k_f6_*) of the serine/threonine kinase PLK1 is indirectly mediated by MPF (S1) resulting in active PLK1P (S2), which is dephosphorylated (*k_r6_*) by cellular PPases resulting in inactive PLK1. Mechanisms occurring in G2/M involving CCNA:CDK2- and AURKA-dependent phosphorylation are not included in the model and will be accounted for in future studies. The steady-state level of total PLK1 is regulated by constant synthesis (*k_s12_*) of PLK1 and self-degradation (*k_d11.1_*, *k_d12.1_*) and enzymatic degradation mediated by APC/CT:CDH1 (*k_d11.3_*, *k_d12.3_*) of both PLK1 and PLK1P resulting in oscillation.

**(7)**

$$k_{r7} = k'_{r7}\left[\text{MPF}\right]$$

Multiple cellular PPases participate in regulating mitosis by acting in a complex hierarchical organization. Human cellular PPases CDC14A and PP2A:B55 are accounted for in this model. Phosphorylation (*k_r7_*) by MPF (S1) inactivates PPases, while dephosphorylation (*k_f7_*) activates PPases. Reactions involving CDC25C phosphatase are defined in Reactions 2 and 3. The steady-state level of total PPase is regulated by constant synthesis (*k_s13_*) of PPase and self-degradation (*k_d13.1_, k_d14.1_*) of both PPase and PPaseP resulting in oscillation.

**(8)**

APC/C is a nineteen-subunit E3 ubiquitin ligase complex and is stimulated by MPF (S1)- and/or PLK1 (S2)-mediated phosphorylation (*k_f8_*) of subunits APC1 and APC3 resulting in active APC/CP (Reaction 8(i)). Phosphorylation stimulates its association with the unphosphorylated activator protein CDC20 (Reaction 10). Dephosphorylation (*k_r8_*) of APC/CP involves cellular PPases including PP2A:B55. Reaction 8(ii) is analogous to Reaction 8(i) in which APC/C of APC/C:CDH1 is phosphorylated (*k_f8_*) to result in APC/CP:CDH1, while APC/CP of APC/CP: CDH1 is dephosphorylated (*k_r8_*) to result in APC/C:CDH1. In contrast, in Reaction 8(iii), APC/CP of APC/CP:CDC20 is dephosphorylated (*k_r8_*) and is subsequently dissociated from the complex to result in APC/C and CDC20. Association of APC/C and CDC20 is not favorable. The steady-state level of total APC/C is regulated by constant synthesis (*k_s15_*) of APC/C and self-degradation (*k_d15.1_, k_d16.1_*) of both APC/C and APC/CP resulting in oscillation.

**(9)**

$$k_{r9} = k'_{r9}\left[\text{MPF}\right]+k''_{r9}\left[\text{PLK1P}\right]; k_{f9} = k'_{f9}\left[\text{PPase}\right]$$

In Reaction 9(i), CDC20 is phosphorylated (*k_r9_*) by MPF (S1) and/or PLK1 (S2) resulting in inactive CDC20P. Dephosphorylation (*k_f9_*) of CDC20P is mediated by cellular PPases including PP2A:B55. In Reaction 9(ii), CDC20 of APC/CP:CDC20 is phosphorylated (*k_r9_*) and is subsequently dissociated from the complex to result in APC/CP and CDC20P. Association of APC/CP and CDC20P is not favorable. The steady-state level of total CDC20 is regulated by constant synthesis (*k_s17_*) of CDC20 and self-degradation (*k_d17.1_*, *k_d18.1_*) and enzymatic degradation mediated by APC/CT:CDH1 (*k_d17.3_*, *k_d18.3_*) of both CDC20 and CDC20P resulting in oscillation. Mechanisms defining the Spindle Assembly Checkpoint are not considered in this model, and will be accounted for in future studies.

**(10)**

Unphosphorylated activator protein CDC20 associates (*k_f10_*) with APC/CP resulting in an active APC/CP:CDC20 complex. Dissociation (*k_r8_*) occurs upon reductions in CDC20 levels due to enzymatic degradation of both CDC20 and CDC20P mediated by APC/CT:CDH1 (*k_d17.3_, k_d18.3_*).

**(11)**

$$k_{r11} = k'_{r11}\left[\text{MPF}\right]+k''_{r11}\left[\text{PLK1P}\right]; k_{f11} = k'_{f11}\left[\text{PPase}\right]$$

In Reaction 11(i), CDH1 is phosphorylated (*k_r11_*) by MPF (S1) and/or PLK1 (S2) resulting in inactive CDH1P. Dephosphorylation (*k_f11_*) of CDH1P is mediated by cellular PPases including PP2A:B55. In Reactions 11(ii) and 11(iii), CDH1 of both APC/C:CDH1 and APC/CP:CDH1 is phosphorylated (*k_r11_*) and is subsequently dissociated from the respective protein complexes. The respective association reactions are not favorable. CDH1 associates with either APC/C or APC/CP (Reactions 12 and 13). The steady-state level of total CDH1 is regulated by constant synthesis (*k_s20_*) of CDH1 and self-degradation (*k_d20.1_, k_d21.1_*) of both CDH1 and CDH1P resulting in oscillation. Additional mechanisms of regulation occurring in the M to G1 transition are not included in the model and will be considered in future studies.

**(12)**

APC/C E3 ubiquitin ligase complex is activated upon its association (*k_f12_*) with the activator protein CDH1 and is inactivated following its dissociation (*k_r12_*) from APC/C:CDH1 complex. CDH1 activates both APC/C and APC/CP to a similar extent. Dynamic changes in CDH1 phosphorylation (Reaction 11) but not steady-state levels result in oscillation.

**(13)**

Phosphorylated APC/C (APC/CP) is activated upon its association (*k_f13_*) with the activator protein CDH1 and is inactivated following its dissociation (*k_r13_*) from APC/CP:CDH1 complex. As stated above, CDH1 activates both APC/C and APC/CP to a similar extent, and dynamic changes in CDH1 phosphorylation (Reaction 11) but not steady-state levels result in oscillation.

**(14)**

$$k_{r14} = k'_{r14}\left[\text{MPF}\right]; k_{f14} = k'_{f14}\left[\text{PPase}\right]$$

PTTG1 is phosphorylated (*k_r14_*) by MPF (S1) to PTTG1P, which is dephosphorylated (*k_f14_*) by cellular PPases to PTTG1. The steady-state level of total PTTG1 is regulated by constant synthesis (*k_s24_*) of PTTG1 and self-degradation (*k_d24.1_*, *k_d25.1_*) and enzymatic degradation mediated by APC/CP:CDC20 (*k_d24.2_*, *k_d25.2_*) of PTTG1 and PTTG1P resulting in oscillation. The degradation of PTTG1P also involves mediation by the E3 ligase SCF-βTrCP, which is not explicitly accounted for, and is lumped with that by APC/CP:CDC20. During mitosis, the loss of PTTG1 results in the activation of the enzyme ESPL1 (Separase) mediating sister chromatid separation.

**(15)**

$$k_{f15} = k'_{f15}\left[\text{MPF}\right]$$

LMNA is a component of the nuclear lamina located on the inner nuclear membrane and is phosphorylated (*k_f15_*) during mitosis by several kinases including MPF (S1). LMNAP contributes to lamina disassembly. LMNAP dephosphorylation (*k_r15_*) to LMNA involves several mechanisms. Because LMNAP is a readout MPF activity, we have not directly accounted for mechanisms resulting in LMNA.

**References**

1. Tyson JJ. Modeling the cell division cycle: cdc2 and cyclin interactions. Proc Natl Acad Sci U S A. 1991;88(16):7328-32. PubMed PMID: 1831270; PubMed Central PMCID: PMCPMC52288.

2. Novak B, Tyson JJ. Numerical analysis of a comprehensive model of M-phase control in Xenopus oocyte extracts and intact embryos. Journal of cell science. 1993;106 ( Pt 4):1153-68. Epub 1993/12/01. PubMed PMID: 8126097.

3. Sible JC, Tyson JJ. Mathematical modeling as a tool for investigating cell cycle control networks. Methods. 2007;41(2):238-47. doi: 10.1016/j.ymeth.2006.08.003. PubMed PMID: 17189866; PubMed Central PMCID: PMCPMC1993813.

4. Tuck C, Zhang T, Potapova T, Malumbres M, Novak B. Robust mitotic entry is ensured by a latching switch. Biol Open. 2013;2(9):924-31. doi: 10.1242/bio.20135199. PubMed PMID: 24143279; PubMed Central PMCID: PMCPMC3773339.

5. Harper JW, Elledge SJ, Keyomarsi K, Dynlacht B, Tsai LH, Zhang P, et al. Inhibition of cyclin-dependent kinases by p21. Mol Biol Cell. 1995;6(4):387-400. Epub 1995/04/01. PubMed PMID: 7626805; PubMed Central PMCID: PMCPMC301199.
