# Supplementary material for: Network mechanisms and dysfunction within an integrated computational model of progression through mitosis in the human cell cycle
Source: PLoS Comput Biol. 2020 Apr 6;16(4):e1007733. doi: 10.1371/journal.pcbi.1007733 (PMC7162553; doi:10.1371/journal.pcbi.1007733)
Supplement: S3 Appendix — (DOCX) [file pcbi.1007733.s003.docx]

**S3 Appendix: ODEs Governing the Mitotic Cell Cycle Regulation**

This Appendix describes the dynamic integrated computational model of the human mitotic cell cycle, which consists of 26 ODEs (ordinary differential equations) and governs the dynamics of the mitotic proteins and associated protein complexes during mitosis. The ODEs are constructed based on the principle of mass conservation for the mitotic proteins/protein complexes using a hybrid Michaelis-Menten (MM) and mass action kinetic formulation for the mitotic interacting reactions. In these ODEs, [X] denotes the relative concentration of a protein or protein complex X (normalized with respect to the total CDK1 concentration), *k_sn_* denotes the synthesis rate constants, *k_dn.m_* denotes the degradation rate constants, *k_fn_* denotes the forward reaction rate constants (cell cycle acceleration), and *k_rn_* denotes the reverse reaction rate constants (cell cycle deceleration). The degradation occurs via multiple processes (i.e. self and/or enzymatically by APC/CP:CDC20 and/or APC/CT:CDH1), as established experimentally. The mitotic gene and protein names are as listed in S1 Appendix, and the mitotic interacting reactions are as defined in S2 Appendix. Proteins suffixed with P denotes the phosphorylated protein and that suffixed with T denotes the total protein.

The mathematical formulation constitutes a total of 105 kinetic parameters in the model. Values of many of these parameters are directly adopted or fine-tuned from relevant published models in the literature from other laboratories [1-9], and the remaining parameter values are estimated based on scientific reasoning and thermodynamic and kinetic constraints (see S4 Appendix) to reproduce the cardinal features (some quantitatively and some qualitatively) of the human mitotic cell cycle based on recently published data. These studies are detailed in the Results section. To obtain the numerical solutions for the ODEs, we set the initial concentrations for the unphosphorylated proteins (i.e. CDK1, CDC25C, WEE1, PLK1, PPase, APC/C, CDC20, CDH1, PTTG1, and LMNA) to 1 and the corresponding phosphorylated proteins and all other proteins and protein complexes to 0, including CCNB1 and CDKN1A (p21^CIP1^). We note here that the 26 ODEs describe the dynamic alterations in the protein activities (i.e. relative concentrations normalized with respect to the total CDK1 concentration), which are non-dimensional variables (unitless). Integrating these ODEs provides dynamic simulations that characterize the mitotic entry, anaphase transition, and mitotic exit. Additional phases of the cell cycle and the associated protein dynamics as well as intracellular compartmentalization are not considered in this model of the human mitotic cell cycle. Cytosolic and nucleus compartments are lumped into a single cellular compartment.

We present below how 26 ODEs were constructed and what key interacting reactions and mechanisms were considered for constructing these ODEs. The values of the rate constants for the mitotic interacting reactions and protein syntheses and degradations are chosen to have an average duration of the cell cycle as 48 hrs (i.e. the duration for the cell to enter into M phase from late G2 phase and exit from M phase into early G1 phase). Different durations of the cell cycle can be obtained by varying a single parameter (time scaling factor; α) in the governing ODEs: d**Y**/dt = α***f**(**Y**, t, **P**), where **f** is a vector function of the protein activities **Y** = **[X]**, time t, and parameter vector **P**. This equation indicates a constant factor is multiplied to all the rate constants.

We mention here that the cell cycle duration is determined by several biological factors, outside of the cell-cycle signaling network, such as nutrient availability, oxygenation, growth factors, and cell-cell contacts. In our mathematical model, this is controlled by the rate constants of syntheses and degradations of the mitotic proteins and by the forward and reverse rate constants of the interacting reactions in the mitotic biopathway. Correspondingly, these rate constants are implicitly regulated by these biological factors. By proportionally increasing or decreasing the rate constants by a factor **α**, we are able to change the duration of the mitotic cell cycle, as demonstrated in S5 Appendix. We believe, we are lumping the cumulative effects of all those biological factors into a single parameters **α**. We also think that different rate constants can be differentially regulated by these distinct biological factors, giving rise to alterations in the cell cycle duration.

**(1) ODE governing the dynamics of free CCNB1**

***The change*** in free CCNB1 over time ***equals the*** ***sum*** of synthesis (*k_s1_*) of CCNB1 and disassembly (*k_r1_*) of CCNB1 from MPF ***minus the sum*** of assembly (*k_f1_*) of CCNB1 into MPF and multiple degradations of CCNB1. Degradations are the sum of elementary decay (*k_d1.1_*) and decay through enzymatic activities (*k_d1.2_*, *k_d1.3_*) mediated by APC/CP:CDC20 and APC/CT:CDH1. CCNB1T equals the sum of free CCNB1 and CCNB1 found in complex form with MPF, preMPF, and CDKN1A:MPF (if CDKN1A is induced). Mechanisms regulating CCNB1 synthesis in G2 are not explicitly accounted for in the model. However, the model simulations confirm that higher CCNB1 synthesis rate (*k_s1_*) results in higher cell cycle oscillation frequencies [10].

**(2) ODE governing the dynamics of free CDK1**

***The change*** in free CDK1 over time ***equals the*** ***sum*** of disassembly (*k_r1_*) of CDK1 from MPF and release of CDK1 following multiple degradations ***minus the sum*** of assembly (*k_f1_*) of CDK1 into MPF. Release of CDK1 from both MPF and preMPF results from CCNB1 degradations by elementary decay (*k_d3.1_, k_d4.1_*) and decay through enzymatic activities (*k_d3.2_, k_d3.3_, k_d4.2_, k_d4.3_*) mediated by APC/CP:CDC20 and APC/CT:CDH1. CDK1T equals the sum of free CDK1 and CDK1 found in complex form with MPF, preMPF, and CDKN1A:MPF (if CDKN1A is induced). Since CDK1 exists abundantly in the cell, [CDK1T] is constant. Since all protein concentrations are normalized with respect to [CDK1T], the relative [CDK1T] = 1.

**(3) ODE governing the dynamics of MPF**

***The change*** in MPF over time ***equals the*** ***sum*** of formation (*k_f1_*) of MPF from CCNB1 and CDK1, dephosphorylation (*k_f2_*) of preMPF to MPF, dissociation (*k_f5_*) of CDKN1A_3_:MPF to MPF and 3 CDKN1A (if induced), and release of MPF following enzymatic degradation (*k_d6.2_*) of CDKN1A_3_:MPF mediated by APC/CP:CDC20 ***minus the sum*** of dissociation (*k_r1_*) of MPF to CCNB1 and CDK1, phosphorylation (*k_r2_*) of MPF to preMPF, formation (*k_r5_*) of CDKN1A_3_:MPF from MPF and 3 CDKN1A (if induced), and multiple degradations of CCNB1 from MPF including elementary decay (*k_d3.1_*) and enzymatic degradations (*k_d3.2_*, *k_d3.3_*) mediated by APC/CP:CDC20 and APC/CT:CDH1. The kinetics of inhibition of MPF activity by CDKN1A indicates a 1 to 3 ratio [11], as described in the Results section. The analysis of kinetic data on MPF and CDKN1A binding also provided the estimate of the binding constant *K_5_* = *k_r5_*/*k_f5_* which provides thermodynamic constraint for the rate constants *k_r5_* and *k_f5_*. The regulations of dephosphorylation (*k_f2_*) of preMPF to MPF by CDC25CP ($k'_{f2}$) and CDC25C ($k''_{f2}$; negligible) and phosphorylation (*k_r2_*) of MPF to preMPF by WEE1 ($k'_{r2}$) and WEE1P ($k''_{r2}$; negligible) are as defined in the S2 Appendix (Reaction 2), following the formulation by Tyson and coworkers [12, 13].

**(4) ODE governing the dynamics of preMPF**

***The change*** in preMPF over time ***equals the*** ***sum*** of phosphorylation (*k_r2_*) of MPF to preMPF involving regulations by WEE1 ($k'_{r2}$) and WEE1P ($k''_{r2}$; negligible) ***minus the sum*** of dephosphorylation (*k_f2_*) of preMPF to MPF involving regulations by CDC25CP$(k'_{f2})$and CDC25C ($k''_{f2}$; negligible), and multiple degradations of CCNB1 from preMPF including elementary decay (*k_d4.1_*) and enzymatic degradations (*k_d4.2_*, *k_d4.3_*) mediated by APC/CP:CDC20 and APC/CT:CDH1.

**(5) ODE governing the dynamics of CDKN1A**

$$\frac{1}{\alpha}\frac{d\left[\text{CDKN1A}\right]}{dt} = k_{s5}+k_{f5}\left[\text{CDKN1A}_{3}\text{:MPF}\right]-k_{r5}\left[\text{MPF}\right]\left[\text{CDKN1A}\right]^{3}- k_{d5.1}\left[\text{CDKN1A}\right]$$

***The change*** in free CDKN1A (p21^CIP1^) over time ***equals the*** ***sum*** of synthesis (*k_s5_*) of CDKN1A and dissociation (*k_f5_*) of 3 CDKN1A from CDKN1A_3_:MPF ***minus the sum*** of association (*k_r5_*) of MPF with 3 CDKN1A and elementary degradation (*k_d5.1_*) of CDKN1A. The kinetics of inhibition of MPF activity by CDKN1A indicates a 1 to 3 ratio [11], and the analysis of kinetic data on MPF and CDKN1A binding provides the estimates of the rate constants *k_r5_* and *k_f5_*.

**(6) ODE governing the dynamics of CDKN1A_3_:MPF**

$$\begin{matrix} \frac{1}{\alpha}\frac{d\left[\text{CDKN1A}_{3}\text{:MPF}\right]}{dt} = k_{r5}\left[\text{MPF}\right]\left[\text{CDKN1A}\right]^{3}-k_{f5}\left[\text{CDKN1A}_{3}\text{:MPF}\right] \\ - k_{d6.2}\left(\frac{\left[\text{APC/CP:CDC20}\right]}{K_{\text{CDKN1A}_{3}\text{:MPF}}+\left[\text{CDKN1A}_{3}\text{:MPF}\right]}\right)\left[\text{CDKN1A}_{3}\text{:MPF}\right] \end{matrix}$$

***The change*** in CDKN1A_3_:MPF complex over time ***equals the*** ***sum*** of association (*k_r5_*) of MPF and 3 CDKN1A ***minus the sum*** of dissociation (*k_f5_*) of CDKN1A_3_:MPF and enzymatic degradation (*k_d6.2_*) of CDKN1A in CDKN1A_3_:MPF (release of MPF) by APC/CP:CDC20.

**(7) ODE governing the dynamics of CDC25CP**

***The change*** in CDC25CP over time ***equals the*** ***sum*** of phosphorylation (*k_f3_*) of CDC25C mediated by MPF and PLK1P ***minus the sum*** of enzymatic dephosphorylation (*k_r3_*) of CDC25CP mediated by PPase and degradations of CDC25CP by elementary decay (*k_d7.1_*) and enzymatic degradation (*k_d7.3_*) mediated by APC/CT:CDH1. The regulations of phosphorylation (*k_f3_*) of CDC25C by MPF (S1) ($k'_{f3}$) and PLK1P (S2) ($k''_{f3}$) and dephosphorylation (*k_r3_*) of CDC25CP by PPase ($k'_{r3}$) are as defined in S2 Appendix (Reaction3).

**(8) ODE governing the dynamics of CDC25C**

***The change*** in CDC25C over time ***equals the sum*** of synthesis (*k_s8_*) of CDC25C and enzymatic dephosphorylation (*k_r3_*) of CDC25CP mediated by PPase ***minus the sum*** of phosphorylation (*k_f3_*) of CDC25C mediated by MPF and PLK1P and degradations of CDC25C by elementary decay (*k_d8.1_*) and enzymatic degradation (*k_d8.3_*) mediated by APC/CT:CDH1. As stated above, phosphorylation (*k_f3_*) is the sum of kinase activities of MPF (S1) ($k'_{f3}$) and PLK1P (S2) ($k''_{f3}$); dephosphorylation (*k_r3_*) is determined by phosphatase activity of PPase ($k'_{r3}$).

**(9) ODE governing the dynamics of WEE1**

***The change*** in WEE1 over time ***equals the*** ***sum*** of synthesis (*k_s9_*) of WEE1 and enzymatic dephosphorylation (*k_r4_*) of WEE1P mediated by PPase ***minus the sum*** of phosphorylation (*k_f4_*) of WEE1 mediated by MPF and PLK1P and elementary degradation (*k_d9.1_*) of WEE1. The regulations of phosphorylation (*k_f4_*) of WEE1 by MPF (S1) ($k'_{f4}$) and PLK1P (S2) ($k''_{f4}$) and dephosphorylation (*k_r4_*) of WEE1P by PPase ($k'_{r4}$) are as defined in S2 Appendix (Reaction 4).

**(10) ODE governing the dynamics of WEE1P**

$$\frac{1}{\alpha}\frac{d\left[\text{WEE1P}\right]}{dt} = k_{f4}\left[\text{WEE}1\right]-k_{r4}\frac{\left[\text{WEE1P}\right]}{K_{\text{WEE1P}}+\left[\text{WEE1P}\right]}-k_{d10.1}\left[\text{WEE1P}\right]$$

***The change*** in WEE1P over time ***equals the*** ***sum*** of phosphorylation (*k_f4_*) of WEE1 mediated by MPF and PLK1P ***minus the sum*** of enzymatic dephosphorylation (*k_r4_*) of WEE1P mediated by PPase and elementary degradation (*k_d10.1_*) of WEE1P. As mentioned above, phosphorylation (*k_f4_*) is the sum of kinase activities of MPF (S1) ($k'_{f4}$) and PLK1P (S2) ($k''_{f4}$); dephosphorylation (*k_r4_*) is determined by phosphatase activity of PPase ($k'_{r4}$).

**(11) ODE governing the dynamics of PLK1P**

***The change*** in PLK1P over time ***equals the*** ***sum*** of phosphorylation (*k_f6_*) of PLK1 mediated by MPF ***minus the sum*** of enzymatic dephosphorylation (*k_r6_*) of PLK1P mediated by PPase and degradations of PLK1P by elementary decay (*k_d11.1_*) and enzymatic degradation (*k_11.3_*) mediated by APC/CT:CDH1. Mechanisms regulating PLK1 in G2/M are not accounted for in the model influencing the timing and magnitude of PLK1 activities in the simulation. The regulations of phosphorylation (*k_f6_*) of PLK1 by MPF (S1) ($k'_{f6}$) and dephosphorylation (*k_r6_*) of PLK1P by PPase ($k'_{r6}$) are as defined in S2 Appendix (Reaction 6).

**(12) ODE governing the dynamics of PLK1**

***The change*** in PLK1 over time ***equals the*** ***sum*** of synthesis (*k_s12_*) of PLK1 and enzymatic dephosphorylation (*k_r6_*) of PLK1P mediated by PPase ***minus the sum*** of phosphorylation (*k_f6_*) of PLK1 mediated by MPF and degradations of PLK1 by elementary decay (*k_d12.1_*) and enzymatic degradation (*k_d12.3_*) mediated by APC/CT:CDH1. As stated above, phosphorylation (*k_f6_*) is defined by kinase activity ($k'_{f6}$) of MPF (S1); dephosphorylation (*k_r6_*) is determined by phosphatase activity ($k'_{r6}$) of PPase. Mechanisms regulating PLK1 in G2/M are not considered in the model.

**(13) ODE governing the dynamics of PPase**

$$\frac{1}{\alpha}\frac{d\left[\text{PPase}\right]}{dt} = k_{s13}+k_{f7}\left[\text{PPaseP}\right]-k_{r7}\left[\text{PPase}\right]-k_{d13.1}\left[\text{PPase}\right]$$

***The change*** in PPase over time ***equals the*** ***sum*** of synthesis (*k_s13_*) of PPase and dephosphorylation (*k_f7_*) of PPaseP ***minus the sum*** of phosphorylation (*k_r7_*) of PPase mediated by MPF and elementary degradation (*k_d13.1_*) of PPase. The regulations of phosphorylation (*k_r7_*) of PPase by MPF (S1) ($k'_{r7}$) is as defined in S2 Appendix (Reaction 7).

**(14) ODE governing the dynamics of PPaseP:**

***The change*** in PPaseP over time ***equals the sum*** of phosphorylation (*k_r7_*) of PPase mediated by MPF ***minus the sum*** of dephosphorylation (*k_f7_*) of PPaseP and elementary degradation (*k_d14_*) of PPaseP. The phosphorylation (*k_r7_*) is defined by kinase activity ($k'_{r7}$) of MPF (S1).

**(15) ODE governing the dynamics of APC/C**

***The change*** in APC/C over time ***equals the*** ***sum*** of APC/C synthesis (*k_s15_*), PPase-mediated dephosphorylation (*k_r8_*) of APC/CP and APC/CP:CDC20 (the later resulting in APC/C and CDC20), disassembly (*k_r12_*) of APC/C:CDH1 resulting in APC/C and CDH1, and MPF- and PLK1P-mediated phosphorylation (*k_r11_*) of APC/C:CDH1 resulting in APC/C and CDH1P ***minus*** ***the sum*** of MPF- and PLK1P-mediated phosphorylation (*k_f8_*) of APC/C, assembly (*k_f12_*) of APC/C:CDH1 from APC/C and CDH1, and elementary degradation (*k_d15.1_*) of APC/C. The regulations of phosphorylation (*k_f8_, k_r9_, k_r11_*) of APC/C, CDC20 and CDH1 by MPF (S1) and PLK1P (S2) and dephosphorylation (*k_r8_, k_f9_, k_f11_*) of APC/CP, CDC20P and CDH1P by PPase are as defined in S2 Appendix (Reactions 8, 9, and 11). For simplicity, the PPase-mediated dephosphorylation and disassembly (*k_r8_*) of APC/CP:CDC20 is considered to have mass action kinetics [1].

**(16) ODE governing the dynamics of APC/CP**

***The change*** in APC/CP over time ***equals the*** ***sum*** of phosphorylation (*k_f8_*) of APC/C mediated by MPF and PLK1P, disassembly (*k_r9_, k_r10_*) of APC/CP:CDC20, and disassembly (*k_r11_, k_r13_*) of APC/CP:CDH1 ***minus the sum*** of enzymatic dephosphorylation (*k_r8_*) of APC/CP mediated by PPase, assembly (*k_f10_*, *k_f13_*) of APC/CP with CDC20 and CDH1, and elementary degradation (*k_d15.1_*) of APC/CP. As stated above, phosphorylation (*k_f8_, k_r9_, k_r11_*) is the sum of kinase activities of MPF (S1) ($k'_{f8}$, $k'_{r9}$, $k'_{r11}$) and PLK1P (S2) ($k''_{f8}$, $k''_{r9}$, $k''_{r11}$); dephosphorylation (*k_r8_, k_f9_, k_f11_*) is determined by phosphatase activity of PPase ($k'_{r8}$, $k'_{f9}$, $k'_{f11}$).

**(17) ODE governing the dynamics of CDC20**

$$\begin{matrix} \frac{1}{\alpha}\frac{d\left[\text{CDC20}\right]}{dt} = k_{s17}+k_{f9}\frac{\left[\text{CDC20P}\right]}{K_{\text{CDC20P1}}+\left[\text{CDC20P}\right]}-k_{r9}\left[\text{CDC20}\right] \\ +\left(k_{r8}+k_{r10}\right)\left[\text{APC/CP:CDC}20\right]-k_{f10}\left[\text{CDC}20\right]\left[\text{APC/CP}\right] \\ -\left(k_{d17.1}+k_{d17.3}\frac{\left[\text{APC/CT:CDH1}\right]}{K_{\text{CDC20}}+\left[\text{CDC}20\right]}\right)\left[\text{CDC}20\right] \end{matrix}$$

***The change*** in CDC20 over time ***equals the*** ***sum*** of synthesis (*k_s17_*) of CDC20, PPase-mediated dephosphorylation (*k_f9_*) of CDC20P, and disassembly (*k_r8_, k_r10_*) of APC/CP:CDC20 (one to APC/C and CDC20 due to dephosphorylation of APC/CP and the other to APC/CP and CDC20) ***minus*** ***the sum*** of MPF- and PLK1P-mediated phosphorylation (*k_r9_*) of CDC20, assembly (*k_f10_*) of CDC20 with APC/CP to form APC/CP:CDC20, and degradations of CDC20 by elementary decay (*k_d17.1_*) and enzymatic degradation (*k_d17.3_*) mediated by APC/CT:CDH1.

**(18) ODE governing the dynamics of CDC20P**

$$\begin{matrix} \frac{1}{\alpha}\frac{d\left[\text{CDC20P}\right]}{dt} = k_{r9}\left[\text{CDC20}\right]-k_{f9}\frac{\left[\text{CDC20P}\right]}{K_{\text{CDC20P1}}+\left[\text{CDC20P}\right]}+ k_{r9}\left[\text{APC/CP}:\text{CDC20}\right] \\ -\left(k_{d18.1}+k_{d18.3}\frac{\left[\text{APC/CT:CDH1}\right]}{K_{\text{CDC20P2}}+\left[\text{CDC20P}\right]}\right)\left[\text{CDC20P}\right] \end{matrix}$$

***The change*** in CDC20P over time ***equals the*** ***sum*** of MPF- and PLK1P-mediated phosphorylation (*k_r9_*) of CDC20 and disassembly (*k_r9_*) of APC/CP:CDC20 by phosphorylation of CDC20 ***minus*** ***the sum*** of PPase-mediated dephosphorylation (*k_f9_*) of CDC20P and degradations of CDC20P by elementary decay (*k_d18.1_*) and enzymatic degradation (*k_d18.2_*) mediated by APC/CT:CDH1.

**(19) ODE governing the dynamics of APC/CP:CDC20**

$$\frac{1}{\alpha}\frac{d\left[\text{APC/CP:CDC}20\right]}{dt} = k_{f10}\left[\text{APC/CP}\right]\left[\text{CDC}20\right]-(k_{r8}+k_{r9}+k_{r10})\left[\text{APC/CP:CDC}20\right]$$

***The change*** in APC/CP:CDC20 complex over time ***equals the*** ***sum*** of assembly (*k_f10_*) of APC/CP:CDC20 from APC/CP and CDC20 ***minus the sum*** of disassembly (*k_r8_, k_r9_, k_r10_*) of APC/CP:CDC20 to different components mediated by PPase (dephosphorylation of APC/CP; *k_r8_*) or MPF and PLK1P (phosphorylation of CDC20; *k_r9_*).

**(20) ODE governing the dynamics of CDH1**

$$\begin{matrix} \frac{1}{\alpha}\frac{d\left[\text{CDH1}\right]}{dt} = k_{s20}-k_{r11}\left[\text{CDH1}\right]+k_{f11}\frac{\left[\text{CDH1P}\right]}{K_{\text{CDH1P}}+\left[\text{CDH1P}\right]} \\ +k_{r12}\left[\text{APC/C:CDH1}\right]-k_{f12}\left[\text{CDH1}\right]\left[\text{APC/C}\right] \\ + k_{r13}\left[\text{APC/CP}:\text{CDH1}\right]- k_{f13}\left[\text{CDH1}\right]\left[\text{APC/CP}\right]-k_{d20.1}\left[\text{CDH1}\right] \end{matrix}$$

***The change*** in CDH1 over time ***equals the*** ***sum*** of synthesis (*k_s20_*) of CDH1, enzymatic dephosphorylation (*k_f11_*) of CDH1P mediated by PPase, and disassemblies (*k_r12_, k_r13_*) of both APC/C:CDH1 and APC/CP:CDH1 ***minus the sum*** of assemblies (*k_f12_, k_f13_*) of CDH1 with APC/C and APC/CP and self-degradation (*k_d20_*) of CDH1. Mechanisms of CDH1 inactivation occurring in G1 are not accounted for in this model.

**(21) ODE governing the dynamics of CDH1P**

***The change*** in CDH1P over time ***equals the*** ***sum*** of phosphorylation (*k_r11_*) of CDH1, APC/C:CDH1 and APC/CP:CDH1 mediated by MPF and PLK1P (the latter are disassembled to APC/C, APC/CP and CDH1P upon phosphorylation of CDH1) ***minus the sum*** of enzymatic dephosphorylation (*k_f11_*) of CDH1P mediated by PPase and self-degradation (*k_d21_*) of CDH1P.

**(22) ODE governing the dynamics of APC/C:CDH1**

$$\begin{matrix} \frac{1}{\alpha}\frac{d\left[\text{APC/C}:\text{CDH}1\right]}{dt} = k_{f12}\left[\text{APC/C}\right]\left[\text{CDH}1\right]+k_{r8}\left[\text{APC/CP:CDH1}\right] \\ - (k_{f8}+k_{r11}+k_{r12})\left[\text{APC/C:CDH}1\right] \end{matrix}$$

***The change*** in APC/C:CDH1 complex over time ***equals the*** ***sum*** of assembly (*k_f12_*) of APC/C: CDH1 from APC/C and CDH1 and dephosphorylation (*k_r8_*) of APC/CP:CDH1 ***minus the sum*** of phosphorylation (*k_f8_*) of APC/C:CDH1and disassembly (*k_r11_, k_r12_*) of APC/C:CDH1 (one due to phosphorylation of CDH1; *k_r11_*).

**(23) ODE governing the dynamics of APC/CP:CDH1:**

$$\begin{matrix} \frac{1}{\alpha}\frac{d\left[\text{APC/CP}:\text{CDH}1\right]}{dt} = k_{f13}\left[\text{APC/CP}\right]\left[\text{CDH}1\right]+k_{f8}\left[\text{APC/C:CDH1}\right] \\ - (k_{r8}+k_{r11}+k_{r13})\left[\text{APC/CP:CDH}1\right] \end{matrix}$$

***The change*** in APC/CP:CDH1 complex over time ***equals the*** ***sum*** of assembly (*k_f13_*) of APC/CP: CDH1 from APC/CP and CDH1 and phosphorylation (*k_f8_*) of APC/C:CDH1 ***minus the sum*** of dephosphorylation (*k_r8_*) of APC/CP:CDH1 and disassembly (*k_r11_, k_r13_*) of APC/CP:CDH1 (one due to dephosphorylation of CDH1; *k_r11_*).

**(24) ODE governing the dynamics of PTTG1**

$$\begin{matrix} \frac{1}{\alpha}\frac{d\left[\text{PTTG}1\right]}{dt} = k_{s24}+k_{f14}\frac{\left[\text{PTTG1P}\right]}{K_{\text{PTTG1P1}}+\left[\text{PTTG}1\text{P}\right]}-k_{r14}\left[\text{PTTG1}\right] \\ - \left(k_{d24.1}+k_{d24.2}\frac{\left[\text{APCP:CDC}20\right]}{K_{\text{PTTG1}}+\left[\text{PTTG}1\right]}\right)\left[\text{PTTG}1\right] \end{matrix}$$

***The change*** in PTTG1 over time ***equals the*** ***sum*** of synthesis (*k_s24_*) of PTTG1 and enzymatic dephosphorylation (*k_f14_*) of PTTG1P mediated by PPase ***minus the sum*** of phosphorylation (*k_r14_*) of PTTG1 mediated by MPF and degradations of PTTG1 by elementary decay (*k_d24.1_*) and enzymatic degradation (*k_d24.2_*) mediated by APC/CP:CDC20. Release and activation of ESPL1 (Separase) from PTTG1 is not accounted for in the model. The regulations of phosphorylation (*k_r14_*) of PTTG1 by MPF (S1) ($k'_{r14}$) and dephosphorylation (*k_f14_*) of PTTG1P by PPase ($k'_{f14}$) are as defined in S2 Appendix (Reaction 11).

**(25) ODE governing the dynamics of PTTG1P**

$$\begin{matrix} \frac{1}{\alpha}\frac{d\left[\text{PTTG1P}\right]}{dt} = k_{r14}\left[\text{PTTG1}\right]-k_{f14}\frac{\left[\text{PTTG1P}\right]}{K_{\text{PTTG1P1}}+\left[\text{PTTG}1P\right]} \\ -\left(k_{d25.1}+ k_{d25.2}\frac{\left[\text{APCP}:\text{CDC}20\right]}{K_{\text{PTTG1P2}}+\left[\text{PTTG1P}\right]}\right)\left[\text{PTTG1P}\right] \end{matrix}$$

***The change*** in PTTG1P over time ***equals the*** ***sum*** of phosphorylation (*k_r14_*) of PTTG1 mediated by MPF ***minus the sum*** of enzymatic dephosphorylation (*k_f14_*) of PTTG1P mediated by PPase and degradations of PTTG1P by elementary decay (*k_d25.1_*) and enzymatic degradation (*k_d25.2_*) mediated by APC/CP:CDC20. Phosphorylation (*k_r14_*) is defined by kinase activity ($k'_{r14}$) of MPF (S1); dephosphorylation (*k_f14_*) is determined by phosphatase activity ($k'_{f14}$) of PPase.

**(26) ODE governing the dynamics of LMNAP**

$$\frac{1}{\alpha}\frac{d\left[\text{LMNAP}\right]}{dt} = k_{f15}\left[\text{LMNA}\right]-k_{r15}\left[\text{LMNAP}\right]$$

***The change*** in LMNAP over time ***equals*** the phosphorylation (*k_f15_*) of LMNA mediated by MPF (S1) ***minus*** the dephosphorylation (*k_r15_*) of LMNAP. Since LMNA and LMNAP are neither synthesized nor degraded, the total LNAP concentration remains constant (i.e. relative [LMNAT] = 1). Thus, through mass conservation, [LMNA] = 1 – [LMNAP]. The regulation of phosphorylation (*k_f15_*) of LMNA by MPF (S1) ($k'_{f15}$) is as defined in S2 Appendix (Reaction 15).

**References**

1. Ciliberto A, Lukacs A, Toth A, Tyson JJ, Novak B. Rewiring the exit from mitosis. Cell Cycle. 2005;4(8):1107-12. PubMed PMID: 15970669.

2. Gerard C, Tyson JJ, Novak B. Minimal models for cell-cycle control based on competitive inhibition and multisite phosphorylations of Cdk substrates. Biophys J. 2013;104(6):1367-79. doi: 10.1016/j.bpj.2013.02.012. PubMed PMID: 23528096; PubMed Central PMCID: PMCPMC3602763.

3. Tyson JJ. Modeling the cell division cycle: cdc2 and cyclin interactions. Proc Natl Acad Sci U S A. 1991;88(16):7328-32. PubMed PMID: 1831270; PubMed Central PMCID: PMCPMC52288.

4. Tyson JJ, Novak B. Regulation of the eukaryotic cell cycle: molecular antagonism, hysteresis, and irreversible transitions. J Theor Biol. 2001;210(2):249-63. doi: 10.1006/jtbi.2001.2293. PubMed PMID: 11371178.

5. Verdugo A, Vinod PK, Tyson JJ, Novak B. Molecular mechanisms creating bistable switches at cell cycle transitions. Open Biol. 2013;3(3):120179. doi: 10.1098/rsob.120179. PubMed PMID: 23486222; PubMed Central PMCID: PMCPMC3718337.

6. Vinod PK, Zhou X, Zhang T, Mayer TU, Novak B. The role of APC/C inhibitor Emi2/XErp1 in oscillatory dynamics of early embryonic cell cycles. Biophys Chem. 2013;177-178:1-6. doi: 10.1016/j.bpc.2013.03.002. PubMed PMID: 23562861.

7. Gérard C, Goldbeter A. Temporal self-organization of the cyclin/Cdk network driving the mammalian cell cycle. Proceedings of the National Academy of Sciences. 2009;106(51):21643. doi: 10.1073/pnas.0903827106.

8. Passos JF, Nelson G, Wang C, Richter T, Simillion C, Proctor CJ, et al. Feedback between p21 and reactive oxygen production is necessary for cell senescence. Mol Syst Biol. 2010;6:347. doi: 10.1038/msb.2010.5. PubMed PMID: 20160708; PubMed Central PMCID: PMCPMC2835567.

9. Tsai TY, Theriot JA, Ferrell JE, Jr. Changes in oscillatory dynamics in the cell cycle of early Xenopus laevis embryos. PLoS Biol. 2014;12(2):e1001788. doi: 10.1371/journal.pbio.1001788. PubMed PMID: 24523664; PubMed Central PMCID: PMCPMC3921120.

10. Tsai TY, Choi YS, Ma W, Pomerening JR, Tang C, Ferrell JE, Jr. Robust, tunable biological oscillations from interlinked positive and negative feedback loops. Science. 2008;321(5885):126-9. Epub 2008/07/05. doi: 10.1126/science.1156951. PubMed PMID: 18599789; PubMed Central PMCID: PMCPMC2728800.

11. Harper JW, Elledge SJ, Keyomarsi K, Dynlacht B, Tsai LH, Zhang P, et al. Inhibition of cyclin-dependent kinases by p21. Mol Biol Cell. 1995;6(4):387-400. Epub 1995/04/01. PubMed PMID: 7626805; PubMed Central PMCID: PMCPMC301199.

12. Novak B, Tyson JJ. Numerical analysis of a comprehensive model of M-phase control in Xenopus oocyte extracts and intact embryos. Journal of cell science. 1993;106 ( Pt 4):1153-68. Epub 1993/12/01. PubMed PMID: 8126097.

13. Sible JC, Tyson JJ. Mathematical modeling as a tool for investigating cell cycle control networks. Methods. 2007;41(2):238-47. doi: 10.1016/j.ymeth.2006.08.003. PubMed PMID: 17189866; PubMed Central PMCID: PMCPMC1993813.
