# Supplementary material for: Network mechanisms and dysfunction within an integrated computational model of progression through mitosis in the human cell cycle
Source: PLoS Comput Biol. 2020 Apr 6;16(4):e1007733. doi: 10.1371/journal.pcbi.1007733 (PMC7162553; doi:10.1371/journal.pcbi.1007733)
Supplement: S4 Appendix — (DOCX) [file pcbi.1007733.s004.docx]

**S4 Appendix: Kinetic Parameters of the Model**

This Appendix describes the kinetic parameters of the model, their definitions, their values used in the model simulations, and the associated references. The mathematical formulations in S2 Appendix and S3 Appendix constitutes a total of 105 kinetic parameters in the model. Values of many of these parameters are directly adopted (Class 1 parameters; C1) or fine-tuned (Class 2 parameters; C2) from relevant published models in the literatures [1-9], and the remaining parameter values (Class 3 parameters; C3) are estimated based on scientific reasoning and thermodynamic and kinetic constraints (see S2 Appendix and S3 Appendix) to reproduce the cardinal features (some quantitatively and some qualitatively) of the human mitotic cell cycle based on recently published data, as detailed in the Results section. The assumptions in the model parameterization include the followings:

1. The synthesis rate constants for most of the unphosphorylated proteins (CCNB1, CDC25C, WEE1, PLK1, APC/C, CDC20, CDH1, PPase, and PTTG1) are set to a value of 0.1 1/hr. The synthesis rate constant for CDKN1A (p21^CIP1^) is negligible (0.0001 1/hr) under normal conditions.
2. Out of 8 mitotic proteins, 4 inactive proteins (CDC25C, WEE1P, APC/C, and CDH1P) are assumed to be self-degraded faster than the corresponding active ones (CDC25CP, WEE1, APC/CP, and CDH1). For the remaining 4 proteins (PLK1, PTTG1, CDC20, and PPase), the active protein degradation rates are the same as the inactive protein degradation rates.
3. The self-degradation rate constants for proteins are usually set at 0.1 1/hr or 0.2 1/hr, except those for preMPF and MPF. On the other hand, the APC/C-mediated degradation rate constants for proteins are usually set to 1 1/hr or 2 1/hr (usually an order of magnitude higher than the self-degradation rate constants), except those of CDC25C and CDC25CP.
4. The Michaelis-Menten constants for the substrates for all enzymatic dephosphorylation reactions mediated by PPase and enzymatic degradations mediated by APC/CP:CDC20 and APC/CT:CDH1 are set to 0.1 (1/10^th^ of the maximal relative substrate concentrations).
5. The association rate constants are usually one order of magnitude higher than the dissociation rate constants for highly favorable reactions (e.g. association of CCNB1 and CDK1 to form MPF; association of APC/CP and CDC20 to form APC/CP:CDC20).
6. All the association and dissociation rate constants were initially set to 80:20 ratio [1]. However, in the case of CDKN1A:MPF, it was adjusted to an 80:8 ratio [10]. In the case of APC/CP:CDH1, it was adjusted to an 80:3 ratio, according to experimental data [11].
7. To determine the phosphorylation and dephosphorylation rates of APC/C and CDC20, we used the phosphorylation and dephosphorylation rates of CDH1 as constraint. Hein *et al.* [12] suggest that the dephosphorylation rates of APC/C and CDH1 are very low compared to that of CDC20. However, we found that the phosphorylation rates of APC/C and CDC20 have no constraints in our model except balancing the strengths of their dephosphorylation rates (thermodynamic and kinetic constraints). We have only one constraint that the phosphorylation rate of CDH1 should be large. Because it is likely that the characteristics of CDC20 and CDH1 would be similar, the phosphorylation and dephosphorylation rates of CDC20 should also be large. Hence, the phosphorylation and dephosphorylation rates of APC/C, CDC20 and CDH1 are set suitably.
8. Without giving larger signal strengths for PLK1 than those for MPF in regulating CDC25C, WEE1 and APC/C phosphorylations, we could not obtain full activity of APC/CP:CDC20 and APC/C:CDH1. We concluded that MPF is indispensable for mitosis progression and cell cycle duration, whereas PLK1 is likely to be the major regulatory signal for mitosis progression.
9. The initial model parameter values are adopted from the frog mitotic cell cycle model of Tyson group [13]. We borrowed their estimates of ratios of the forward and backward rate constants, assuming that the hypothetical rate constant ratios are universal in all organisms [14]. We also considered many other published literatures in basing our model parameter values [1-9].

**Parameter values of the model**

| **Parameter** | **Definition** | **Values** | **Reference** |
| --- | --- | --- | --- |
| $k_{f1}$ | Forward rate constant for association of CCNB1 and CDK1 | 18 | C2 [3] |
| $k_{r1}$ | Reverse rate constant for dissociation of MPF | 0.1 | C2 [3] |
| $k'_{f2}$ | Dephosphorylation rate constant of preMPF by CDC25CP | 10 | C1 [1, 9] |
| $k''_{f2}$ | Dephosphorylation rate constant of preMPF by CDC25C | 0.2 | C1[1] |
| $k'_{r2}$ | Phosphorylation rate constant of MPF by WEE1 | 4 | C1 [1, 9] |
| $k''_{r2}$ | Phosphorylation rate constant of MPF by WEE1P | 0.1 | C1 [1] |
| $k'_{f3}$ | Phosphorylation rate constant of CDC25C by MPF | 1 | C1 [1] |
| $k''_{f3}$ | Phosphorylation rate constant of CDC25C by PLK1P | 3 | C3 [7] |
| $k'_{r3}$ | Dephosphorylation rate constant of CDC25CP by PPase | 0.2 | C1 [1] |
| $k'_{f4}$ | Phosphorylation rate constant of WEE1 by MPF | 1 | C1 [1] |
| $k''_{f4}$ | Phosphorylation rate constant of WEE1 by PLK1P | 3 | C3 |
| $k'_{r4}$ | Dephosphorylation rate constant of WEE1 by PPase | 0.2 | C1 [1] |
| $k_{f5}$ | Forward rate constant for dissociation of CDKN1A:MPF | 8 | C3 [10] |
| $k_{r5}$ | Reverse rate constant for association of CDKN1A and MPF | 80 | C3 [10] |
| $k'_{f6}$ | Phosphorylation rate constant of PLK1 by MPF | 5 | C3 |
| $k'_{r6}$ | Dephosphorylation rate constant of PLK1P | 0.5 | C3 |
| $k_{f7}$ | Dephosphorylation rate constant of PPaseP | 0.5 | C2 [6] |
| $k_{r7}$ | Phosphorylation rate constant of PPase by MPF | 4 | C2 [6] |
| $k'_{f8}$ | Phosphorylation rate constant of APC/C by MPF | 0.1 | C2 [1] |
| $k''_{f8}$ | Phosphorylation rate constant of APC/C by PLK1P | 0.3 | C3 |
| $k'_{r8}$ | Dephosphorylation rate constant of APC/CP by PPase | 0.04 | C1 [1] |
| $k'_{r9}$ | Phosphorylation rate constant of CDC20 by MPF | 12 | C2 [1] |
| $k''_{r9}$ | Phosphorylation rate constant of CDC20 by PLK1P | 12 | C3 |
| $k'_{f9}$ | Dephosphorylation rate constant of CDC20 by PPase | 20 | C3 [12] |
| $k_{f10}$ | Forward rate constant for association of APC/CP and CDC20 | 80 | C1 [1] |
| $k_{r10}$ | Reverse rate constant for dissociation of APC/CP:CDC20 | 20 | C1 [1] |
| $k'_{f11}$ | Dephosphorylation rate constant of CDH1P by PPase | 0.01 | C3 [12] |
| $k'_{r11}$ | Phosphorylation rate constant of CDH1 by MPF | 24 | C3 |
| $k''_{r11}$ | Phosphorylation rate constant of CDH1 by PLK1 | 24 | C3 |
| $k_{f12}$ | Forward rate constant for association of APC/C and CDH1 | 80 | C2 [1, 11] |
| $k_{r12}$ | Reverse rate constant for dissociation of APC/C:CDH1 | 20 | C2 [1, 11] |
| $k_{f13}$ | Forward rate constant for association of APC/CP and CDH1 | 80 | C2 [1, 11] |
| $k_{r13}$ | Reverse rate constant for dissociation of APC/CP:CDH1 | 3 | C2 [1, 11] |
| $k'_{f14}$ | Dephosphorylation rate constant of PTTG1P by PPase | 0.01 | C3 |
| $k'_{r14}$ | Phosphorylation rate constant of PTTG1 by MPF | 5 | C3 |
| $k'_{f15}$ | Phosphorylation rate constant of LMNA by MPF | 1 | C3 |
| $k_{r15}$ | Dephosphorylation rate constant of LMNAP | 1 | C3 |
| $k_{s1}$ | CCNB1synthesis rate constant | 0.1 | C2 [1, 5] |
| $k_{s5}$ | CDKN1A synthesis rate constant | 0.0001 | C2 [8, 10] |
| $k_{s8}$ | CDC25C synthesis rate constant | 0.1 | C2 [7] |
| $k_{s9}$ | WEE1 synthesis rate constant | 0.1 | C2 [7] |
| $k_{s12}$ | PLK1 synthesis rate constant | 0.1 | C3 |
| $k_{s13}$ | PPase synthesis rate constant | 0.1 | C3 |
| $k_{s15}$ | APC/C synthesis rate constant | 0.1 | C3 |
| $k_{s17}$ | CDC20 synthesis rate constant | 0.1 | C2 [7] |
| $k_{s20}$ | CDH1 synthesis rate constant | 0.1 | C2 [7] |
| $k_{s24}$ | PTTG1 synthesis rate constant | 0.1 | C2 [2] |
| $k_{d1.1}$ | CCNB1 self-degradation | 0.1 | C2 [7] |
| $k_{d1.2}$ | CCNB1 degradation by APC/CP:CDC20 | 1 | C2 [1, 5] |
| $k_{d1.3}$ | CCNB1 degradation by APC/CT:CDH1 | 1 | C2 [4, 5] |
| $k_{d3.1}$ | MPF self-degradation | 0.01 | C2 [7] |
| $k_{d3.2}$ | MPF degradation by APC/CP:CDC20 | 1 | C2 [1, 5] |
| $k_{d3.3}$ | MPF degradation by APC/CT:CDH1 | 1 | C2 [4, 5] |
| $k_{d4.1}$ | preMPF self-degradation | 0.01 | C2 [7] |
| $k_{d4.2}$ | preMPF degradation by APC/CP:CDC20 | 1 | C2 [1, 5] |
| $k_{d4.3}$ | preMPF degradation by APC/CT:CDH1 | 1 | C2 [4, 5] |
| $k_{d5.1}$ | CDKN1A self-degradation | 1 | C2 [8] |
| $k_{d6.2}$ | CDKN1A degradation in CDKN1A:MPF by APC/CP:CDC20 | 1 | C3 [1, 8] |
| $k_{d7.1}$ | CDC25CP self-degradation | 0.1 | C2 [7] |
| $k_{d7.3}$ | CDC25CP degradation by APCT:CDH1 | 0.1 | C3 [4, 5] |
| $k_{d8.1}$ | CDC25C self-degradation | 0.2 | C2 [7] |
| $k_{d8.3}$ | CDC25C degradation by APCT:CDH1 | 0.1 | C3 [4, 5] |
| $k_{d9.1}$ | WEE1 self-degradation | 0.1 | C2 [7] |
| $k_{d10.1}$ | WEE1P self-degradation | 1 | C2 [7] |
| $k_{d11.1}$ | PLK1P self-degradation | 0.1 | C3 [7] |
| $k_{d11.3}$ | PLK1P degradation by APC/CT:CDH1 | 2 | C3 [4, 5] |
| $k_{d12.1}$ | PLK1 self-degradation | 0.1 | C3 [7] |
| $k_{d12.3}$ | PLK1 degradation by APC/CT:CDH1 | 2 | C3 [4, 5] |
| $k_{d13.1}$ | PPase self-degradation | 0.1 | C3 [7] |
| $k_{d14.1}$ | PPaseP self-degradation | 0.1 | C3 [7] |
| $k_{d15.1}$ | APC/C self-degradation | 0.2 | C3 [7] |
| $k_{d16.1}$ | APC/CP self-degradation | 0.1 | C3 [7] |
| $k_{d17.1}$ | CDC20 self-degradation | 0.1 | C2 [7] |
| $k_{d17.3}$ | CDC20 degradation by APC/CT:CDH1 | 2 | C2 [4, 5] |
| $k_{d18.1}$ | CDC20P self-degradation | 0.1 | C2 [7] |
| $k_{d18.3}$ | CDC20P degradation by APC/CT:CDH1 | 2 | C2 [4, 5] |
| $k_{d20.1}$ | CDH1 self-degradation | 0.1 | C2 [7] |
| $k_{d21.1}$ | CDH1P self-degradation | 0.2 | C2 [7] |
| $k_{d24.1}$ | PTTG1 self-degradation | 0.1 | C2 [2] |
| $k_{d24.2}$ | PTTG1 degradation by APC/CP:CDC20 | 1 | C2 [2] |
| $k_{d25.1}$ | PTTG1P self-degradation | 0.1 | C2 [2] |
| $k_{d25.2}$ | PTTG1P degradation by APC/CP:CDC20 | 1 | C2 [2] |
| $K_{\text{CCNB1.1}}$ | MM constant of CCNB1 degradation by APC/CP:CDC20 | 0.1 | C3 [1, 4, 7] |
| $K_{\text{CCNB1.2}}$ | MM constant of CCNB1 degradation by APC/CP:CDH1 | 0.1 | C3 [1, 4, 7] |
| $K_{\text{MPF1}}$ | MM constant of MPF degradation by APC/CP:CDC20 | 0.1 | C3 [1, 4, 7] |
| $K_{\text{MPF2}}$ | MM constant of MPF degradation by APC/CT:CDH1 | 0.1 | C3 [1, 4, 7] |
| $K_{\text{preMPF1}}$ | MM constant of MPF degradation by APC/CP:CDC20 | 0.1 | C3 [1, 4, 7] |
| $K_{\text{preMPF2}}$ | MM constant of MPF degradation by APC/CP:CDH1 | 0.1 | C3 [1, 4, 7] |
| $K_{\text{CDKN1A}_{3}\text{:MPF}}$ | MM constant of CDKN1A_3_:MPF degradation by APC/CP:CDC20 | 0.1 | C3 [1, 4, 7] |
| $K_{\text{CDC25CP1}}$ | MM constant of CDC25CP Dephosphorylation by PPase | 0.1 | C3 [1, 4, 7] |
| $K_{\text{CDC25CP2}}$ | MM constant of CDC25CP degradation by APC/CT:CDH1 | 0.1 | C3 [1, 4, 7] |
| $K_{\text{CDC25C}}$ | MM constant of CDC25C degradation by APC/CT:CDH1 | 0.1 | C3 [1, 4, 7] |
| $K_{\text{WEE1P}}$ | MM constant of WEE1P dephosphorylation by PPase | 0.1 | C3 [1, 4, 7] |
| $K_{\text{PLK1P1}}$ | MM constant of PLK1P dephosphorylation by PPase | 0.1 | C3 [1, 4, 7] |
| $K_{\text{PLK1P2}}$ | MM constant of PLK1P degradation by APC/CT:CDH1 | 0.1 | C3 [1, 4, 7] |
| $K_{\text{PLK1}}$ | MM constant of PLK1 degradation by APC/CT:CDH1 | 0.1 | C3 [1, 4, 7] |
| $K_{\text{APCP}}$ | MM constant of APC/CP dephosphorylation by PPase | 0.1 | C3 [1, 4, 7] |
| $K_{\text{CDC20P1}}$ | MM constant of CDC20P dephosphorylation by PPase | 0.1 | C3 [1, 4, 7] |
| $K_{\text{CDC20}}$ | MM constant of CDC20 degradation by APC/CT:CDH1 | 0.1 | C3 [1, 4, 7] |
| $K_{\text{CDC20P2}}$ | MM constant of CDC20P degradation by APC/CT:CDH1 | 0.1 | C3 [1, 4, 7] |
| $K_{\text{CDH1P}}$ | MM constant of CDH1P dephosphorylation by PPase | 0.1 | C3 [1, 4, 7] |
| $K_{\text{PTTG1P1}}$ | MM constant of PTTG1P dephosphorylation by PPase | 0.1 | C3 [1, 4, 7] |
| $K_{\text{PTTG1}}$ | MM constant of PTTG1 degradation by APC/CP:CDC20 | 0.1 | C3 [1, 4, 7] |
| $K_{\text{PTTG1P2}}$ | MM constant of PTTG1P degradation by APC/CP:CDC20 | 0.1 | C3 [1, 4, 7] |
| α | Time scaling factors governing the mitotic cell cycle duration | 1.4 | C3 |

**(Remark)** C1: Class 1 parameters (directly adopted from the cited published literatures); C2: Class 2 parameters (fine-tuned from the cited published literatures); C3: Class 3 parameters, whose values are estimated in the present study. Rate constants are in the units of 1/hr. Since protein concentrations are expressed relative to the total CDK1T concentration, Michalis-Menten (MM) constants are also expressed in terms of the relative concentrations.

**References**

1. Ciliberto A, Lukacs A, Toth A, Tyson JJ, Novak B. Rewiring the exit from mitosis. Cell Cycle. 2005;4(8):1107-12. PubMed PMID: 15970669.

2. Gerard C, Tyson JJ, Novak B. Minimal models for cell-cycle control based on competitive inhibition and multisite phosphorylations of Cdk substrates. Biophys J. 2013;104(6):1367-79. doi: 10.1016/j.bpj.2013.02.012. PubMed PMID: 23528096; PubMed Central PMCID: PMCPMC3602763.

3. Tyson JJ. Modeling the cell division cycle: cdc2 and cyclin interactions. Proc Natl Acad Sci U S A. 1991;88(16):7328-32. PubMed PMID: 1831270; PubMed Central PMCID: PMCPMC52288.

4. Tyson JJ, Novak B. Regulation of the eukaryotic cell cycle: molecular antagonism, hysteresis, and irreversible transitions. J Theor Biol. 2001;210(2):249-63. doi: 10.1006/jtbi.2001.2293. PubMed PMID: 11371178.

5. Verdugo A, Vinod PK, Tyson JJ, Novak B. Molecular mechanisms creating bistable switches at cell cycle transitions. Open Biol. 2013;3(3):120179. doi: 10.1098/rsob.120179. PubMed PMID: 23486222; PubMed Central PMCID: PMCPMC3718337.

6. Vinod PK, Zhou X, Zhang T, Mayer TU, Novak B. The role of APC/C inhibitor Emi2/XErp1 in oscillatory dynamics of early embryonic cell cycles. Biophys Chem. 2013;177-178:1-6. doi: 10.1016/j.bpc.2013.03.002. PubMed PMID: 23562861.

7. Gérard C, Goldbeter A. Temporal self-organization of the cyclin/Cdk network driving the mammalian cell cycle. Proceedings of the National Academy of Sciences. 2009;106(51):21643. doi: 10.1073/pnas.0903827106.

8. Passos JF, Nelson G, Wang C, Richter T, Simillion C, Proctor CJ, et al. Feedback between p21 and reactive oxygen production is necessary for cell senescence. Mol Syst Biol. 2010;6:347. doi: 10.1038/msb.2010.5. PubMed PMID: 20160708; PubMed Central PMCID: PMCPMC2835567.

9. Tsai TY, Theriot JA, Ferrell JE, Jr. Changes in oscillatory dynamics in the cell cycle of early Xenopus laevis embryos. PLoS Biol. 2014;12(2):e1001788. doi: 10.1371/journal.pbio.1001788. PubMed PMID: 24523664; PubMed Central PMCID: PMCPMC3921120.

10. Harper JW, Elledge SJ, Keyomarsi K, Dynlacht B, Tsai LH, Zhang P, et al. Inhibition of cyclin-dependent kinases by p21. Mol Biol Cell. 1995;6(4):387-400. Epub 1995/04/01. PubMed PMID: 7626805; PubMed Central PMCID: PMCPMC301199.

11. Zhang S, Chang L, Alfieri C, Zhang Z, Yang J, Maslen S, et al. Molecular mechanism of APC/C activation by mitotic phosphorylation. Nature. 2016;533(7602):260-4. Epub 2016/04/28. doi: 10.1038/nature17973. PubMed PMID: 27120157; PubMed Central PMCID: PMCPMC4878669.

12. Hein JB, Hertz EPT, Garvanska DH, Kruse T, Nilsson J. Distinct kinetics of serine and threonine dephosphorylation are essential for mitosis. Nat Cell Biol. 2017;19(12):1433-40. doi: 10.1038/ncb3634. PubMed PMID: 29084198.

13. Novak B, Tyson JJ. Numerical analysis of a comprehensive model of M-phase control in Xenopus oocyte extracts and intact embryos. Journal of cell science. 1993;106 ( Pt 4):1153-68. Epub 1993/12/01. PubMed PMID: 8126097.

14. Nurse P. Universal control mechanism regulating onset of M-phase. Nature. 1990;344:503. doi: 10.1038/344503a0.
