# Supplementary material for: Network mechanisms and dysfunction within an integrated computational model of progression through mitosis in the human cell cycle
Source: PLoS Comput Biol. 2020 Apr 6;16(4):e1007733. doi: 10.1371/journal.pcbi.1007733 (PMC7162553; doi:10.1371/journal.pcbi.1007733)
Supplement: S5 Appendix — (DOCX) [file pcbi.1007733.s005.docx]

**S5 Appendix: Oscillations of all Mitotic Proteins**

**
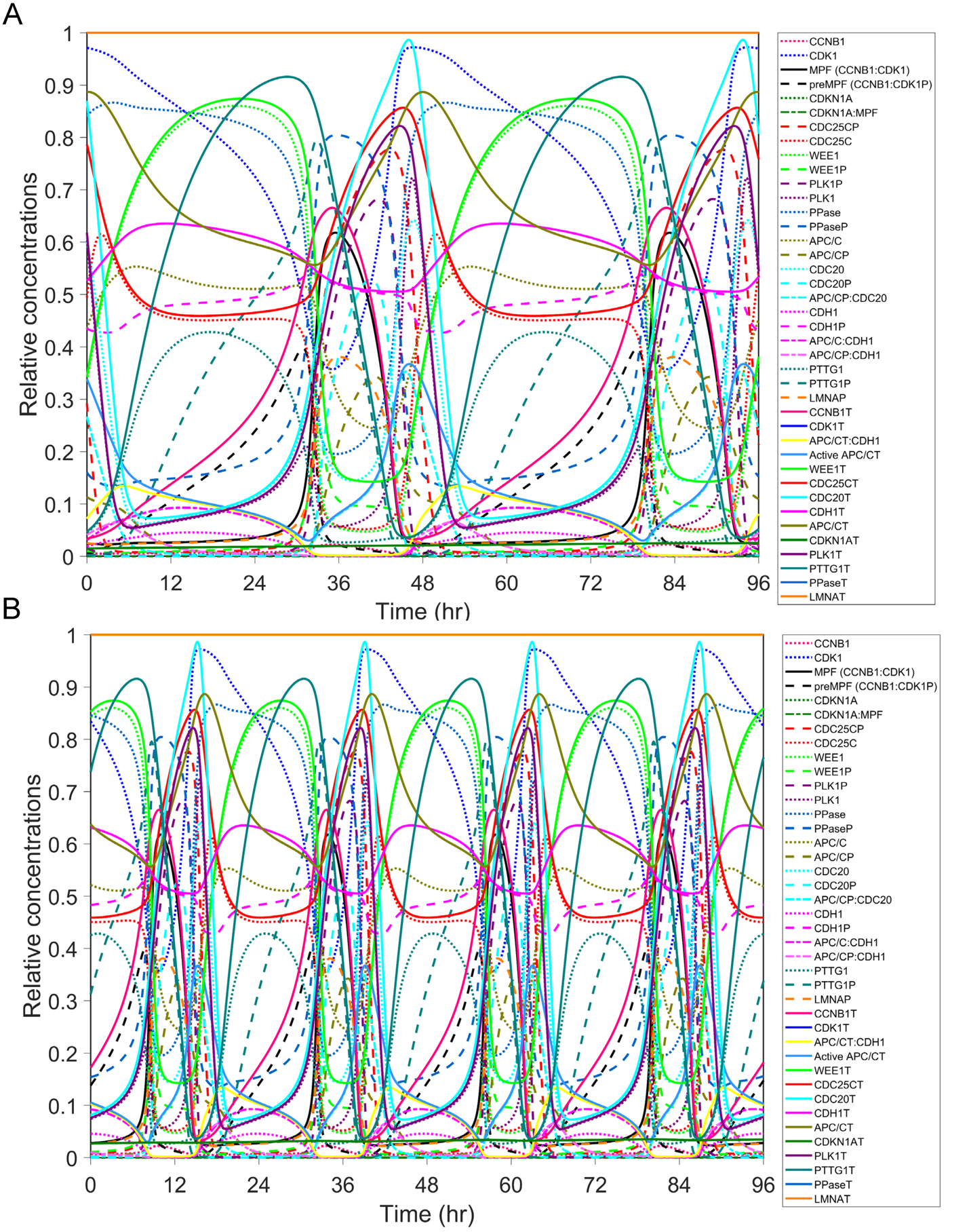
**

**Figure S1. Oscillations of the human mitotic proteins and protein complexes during the progression of cell cycle.** Model simulations show relative concentrations for key mitotic proteins and associated protein complexes during two cycles of mitosis over a **(A)** 48 hr and **(B)** 24 hr time scales, centered on activation/inactivation of MPF (CCNB1:CDK1, solid black line). Total protein or protein complex levels (T, solid lines), phosphorylated proteins (P, wide dashed lines), free unphosphorylated proteins (narrow dashed lines), and regulated interactions (mixed dashed lines) are shown in oscillations relative to each other. Regulations occurring during additional phases of the cell cycle are not accounted for in the model simulation. Mitotic interacting reactions, ordinary differential equations (ODEs), and optimized model parameter values are as presented in S2 Appendix, S3 Appendix, and S4 Appendix, respectively. Simulations in Panels A and B differ in terms of the duration of cell cycle, which is controlled by a single time scale parameter α in the ODEs (S3 Appendix); α = 1.4 for simulations in Panel A, while α = 2.8 for simulations in Panel B.
