# Supplementary material for: Network mechanisms and dysfunction within an integrated computational model of progression through mitosis in the human cell cycle
Source: PLoS Comput Biol. 2020 Apr 6;16(4):e1007733. doi: 10.1371/journal.pcbi.1007733 (PMC7162553; doi:10.1371/journal.pcbi.1007733)
Supplement: Attachment — Submitted filename: ReviewResponse_MitoticCellCycle_02Dec2019.docx [file pcbi.1007733.s006.docx]

**Response to Reviewers’ Comments**

We thank the Editor and Reviewers for the helpful comments and guidance in revising of the manuscript.

**Editor**

1) The authors should describe more explicitly what makes their model unique and especially appropriate for describing human cell cycle regulation.

**RESPONSE:** We have rewritten the abstract and revised the text throughout the manuscript to more explicitly describe the purpose of the model and its unique features (novelty). At the beginning of the abstract we now state, “The cellular protein-protein interaction network that governs cellular proliferation (cell cycle) is highly complex. Here, we have developed a novel computational model of human mitotic cell cycle, integrating diverse cellular mechanisms, for the purpose of generating new hypotheses and predicting new experiments designed to help understand complex diseases. The pathogenic state investigated is infection by a human herpesvirus. The model starts at mitotic entry initiated by the activities of Cyclin-dependent kinase 1 (CDK1) and Polo-like kinase 1 (PLK1), transitions through Anaphase-promoting complex (APC/C) bound to Cell division cycle protein 20 (CDC20), and ends upon mitotic exit mediated by APC/C bound to CDC20 homolog 1 (CDH1). It includes syntheses and multiple mechanisms of degradations of the mitotic proteins. Prior to this work, no such comprehensive model of the human mitotic cell cycle existed.” **(p 2, ln 24-33)**. In addition, we have highlighted unique features (novelty) of the model throughout the document using this or a variation of the phrase, “unique to this model” **(p 7, ln 149-150; p 8, ln 163-168; p 29, 617-620)**.

2) They also must provide more evidence for constraining the values of the kinetic parameters in the model by comparison to quantitative data on mRNA and protein levels during progression through the human cell cycle. Nowadays, to make quantitative predictions of the outcome of novel experiments using a mathematical model, it is customary to predict results using an ensemble of "acceptable" sets of rate parameter values (rather than a single "best" set, or, worse still, a set of parameter values many of which are arbitrary).

**RESPONSE:** We did not have enough “high-resolution” quantitative data for fitting the model to the data and obtain accurate estimates of all the model parameters that best-fit the data. Rather, we used a heuristic approach that is common in the cell cycle modeling field. We have further justified our model parameterization approach throughout the manuscript (e.g. **p 13, ln 272-285; p 38, ln 831-841; Supplemental Information S4 Appendix**). Specifically, values of many of the model parameters are directly adopted (Class 1 parameters) or fine-tuned (Class 2 parameters) from relevant published models in the literatures, and the remaining parameter values (Class 3 parameters) are estimated based on scientific reasoning and thermodynamic and kinetic constraints. This approach has enabled us to reproduce the cardinal features (some quantitatively and some qualitatively) of the human mitotic cell cycle function and dysfunction based on recently published experimental data with diverse cellular perturbations. This is our “selling” point, and we believe the parameter set is not “arbitrary”, as arbitrary parameter values will not describe all the features of the model, consistent with many available “low-resolution” qualitative data on human mitotic cell cycle biology and pathobiology.

3) The authors should address the question (in the discussion?) of what specific aspects of the general model are relevant to the issue of response to viral infection. The authors should also consider other applications of the model to justify the utility of their comprehensive model.

**RESPONSE:** Thank you for identifying this deficiency. We have more precisely stated the question being investigated regarding viral infection (e.g. “the reason for APC/C inactivation and how it contributes to HCMV replication remain unknown” **p 26, ln 573-574**) and the hypothesis generated using this novel model, “By applying this integrated model of mitosis, our simulations have generated the hypothesis that HCMV-mediated inactivation of APC/C is necessary for producing a unique mitotic collapse defined by elevated levels of MPF kinase activity and LMNAP which is necessary for virion nuclear egress.” **(p 27, ln 594-597)**. In addition, we have extended the discussion to aspects of the model relevant to HCMV infection (**p. 32-33, ln 697-716**). Finally, we have discussed a second application of the model. “Our integrated computational model of the mitotic cell cycle will allow for in silico studies that investigate combinatorial approaches limiting MM proliferation and possibly inducing a mitotic collapse and/or catastrophe prior to extensive in vitro experimentation.” **(p 32, ln 691-697)**.

**Reviewer #1**

1) Your model is able to simulate Potapova experiments showing the impact of CDC25C and WEE1 regulation on MPF (Fig. 5). A previous mathematical model already reproduced Potapova experiment (Tuck C. et al. Robust mitotic entry is ensured by a latching switch. Biol. Open 2013). What is the adding value of your model as compared to this previous model? I believe it should be discussed.

**RESPONSE:** We have further clarified the purpose of using the data from Potapova *et al.* in the manuscript and included a statement regarding Tuck *et al.* Specifically, we have added, “Data from Potapova *et al.* [9] was used by Tuck *et al.* [27] to uncover a latching-switch mechanism controlling the G2/M transition during a single cycle. Here, we have tested the model during multiple cycles to reproduce the behaviors experimentally demonstrated by Potapova et al. [3].” (**p 16, ln 349-357**). We also note here that we have used the model to test several other independent experimental data sets, signifying the predictability of our model to reproduce diverse human mitotic cell cycle function under diverse cellular perturbations.

2) You have developed an exhaustive model of the human mitotic cell cycle which incorporates 105 parameters. I am convinced that this approach is useful to better highlight the qualitative dynamics of a regulatory structure, but I am not convinced that you can make strong quantitative predictions. In particular, you predict that the virus-mediated disruption of APC/C is necessary to sustain a unique mitotic collapse. This prediction may be dependent of your specific set of parameter values. The model may exhibit complete different dynamics if you are located in a different region of your parameters space.

**RESPONSE:** Thank you for this reasonable comment. The primary reason of constructing this model was to integrate multiple regulatory mechanisms into a more comprehensive model of mitosis for the purpose of generating new hypotheses and predicting new experiments designed to help understand complex diseases. We did not have enough “high-resolution” quantitative data for fitting the model to the data and obtain accurate estimates of all the model parameters that best-fit the data. Therefore, we used a heuristic approach that is common in the cell cycle modeling field (also see our response to Editor’s 2^nd^ comment above). We believe we have obtained an acceptable parameter set, as the model with these parameter values describes the many cardinal features of the mitotic cell cycle function and dysfunction under diverse cellular perturbations, consistent with recently published experimental data from many laboratories. In fact, this is our “selling” point, and we believe the parameter set is not “arbitrary”, as arbitrary parameters will not describe all the cardinal features of human mitosis. Being a model of a complex system, we do agree with the Reviewer that there might be other parameter sets, which can reproduce the biology, as there will be many parameters correlated with each other.

For a background, this collaborative project began based on our interests to understand how a human herpesvirus manipulates the mitotic network. A large collection of data sets exists that investigated relationship changes during HCMV infection, and these investigators propose diverse mechanisms of action for the many viral proteins involved. During the 72-96 hr viral replication time, viral proteins are altering diverse cellular processes. A more comprehensive model of mitosis and the ability to expand the model will be required to investigate the complex and dynamic mechanisms in network dysregulation during viral infection and other diseases. However, these published data sets compare infected verse uninfected events and we needed to build a more comprehensive simulation to approximate normal mitotic network. Using this initial base model, we will use the data sets comparing uninfected to infected relations to further refine the simulation.

To address this Reviewer’s concerns, we have further clarified the purpose of the model in the manuscript abstract **(p 2, ln 24-33)** and throughout the manuscript, where appropriate **(e.g. p 7, ln 149-150; p 8, ln 163-168; p 29, 617-620)**. We have also extended the discussion specific aspects of the general model as they relate to viral infection (**p. 32-33, ln 698-716**). Although seemingly counter intuitive, we are interested in the outcomes due to possible varying parameter spaces as we advance this work in subsequent studies. Our long term goal is to understand infection and possible outcomes occurring in diverse cellular environments. Finally, we have discussed a second application of the model, studying multiple myeloma. “Our integrated computational model of the mitotic cell cycle will allow for in silico studies that investigate combinatorial approaches limiting MM proliferation and possibly inducing a mitotic collapse and/or catastrophe prior to extensive in vitro experimentation.” **(p 32, ln 691-697)**.

3) You mention that the computational model is for the human mitotic cell cycle because you consider only regulations present in humans. Have you calibrated our model quantitatively on human data? Nowadays, mRNA and protein expression levels of numerous gene can be available for numerous human cancers (i.e. The Cancer Genome Atlas and The Cancer Proteome Atlas: https://tcpaportal.org/tcpa/). This is the same for human cancer cell lines. In addition, half-life durations of mRNAs and proteins can be available in the literature. All the protein data could help you to calibrate your model in a more precise manner, which could reinforce the model’s predictions.

**RESPONSE:** Thank you for your recommendations. We have now investigated several databases including NCBI GEO and the referenced Cancer Proteome Atlas for data sets that would improve the resolution of this model. Unfortunately, we did not find data sets that met our criteria comparing normal relationships overtime. Most data sets compared a single normal data point to multiple points during perturbations or multiple specimens in the absence of kinetic data. The model developed in this manuscript recapitulates changes in relationships overtime and built from data sets obtained from synchronized human cells lines and include a perturbation that altered relationships over time. We do recognize that this model is currently at a low resolution and we will need to further refine it in futures studies. As noted above, a large collection of data sets exists from publications that investigated relationship changes during HCMV infection. We will be refining the base model developed in this manuscript in future studies using data sets comparing normal to HCMV-perturbed relationships over time. To address the Reviewer’s concerns, we have now extended the discussion as outlined in the previous response.

4) The wiring diagram in Figure 1 is exhaustive but very complex. In addition to this scheme, I would like to see a more simplified wiring diagram where the regulatory structure of the network, i.e. positive feedback loops, is better highlighted. I do not think that the time series in Figure 2 is very informative. I propose to remove it or to put it in Supplementary Information.

**RESPONSE:** Thank you for the recommendation and we have now included simplified wiring diagrams in the new Figure 1. This includes two diagrams (**new Fig. 1B and C**) based on the more complex network diagram (new Fig. 1A). We have updated the text (**p 9, ln 179-181**) and figure legends accordingly **(p 10, ln 202-205)**. We have also removed the original Fig. 2 and added it to Supplemental Information (**S5 Appendix**) including simulations for both a 48 hr and a 24 hr cycle time (**new Fig. S1**).

**Reviewer #2:**

1) We applaud the approach of trying to build a comprehensive model, but there is also a downside with respect to tractability of the model and whether such a model can generate unique and falsifiable predictions. The latter point needs to be addressed more clearly. We would prefer a reductionist model, if the same physiological behaviors can be achieved via such a simpler model.

**RESPONSE:** Thank you for this reasonable comment, and we agree with you. However, our objective was to construct a model of human mitosis that accounts for the biological processes that are experimentally established, as detailed throughout the manuscript. For the kind of questions addressed in this manuscript or will be addressed in future studies (see our response to Reviewer 1’s 2^nd^ comment), simple reductionist model will not suffice. As detailed in the manuscript, the developed model is able to capture the complex network of the mitotic cycle of human cells under diverse cellular perturbations as established experimentally by many laboratories. For example, the simulation follows the behaviors observed experimentally which includes required conditions to transition through APC/C:CDC20 to APC/C:CDH1. Our approach has resulted in this base quantitative model of low resolution and we will be refining the simulation in future studies to further refine and calibrate the model.

2) Far too many parameters (85 of the 105) are arbitrarily assigned in order to recapitulate cardinal features of mitosis. This brings the validity of the model into question. Further justification for these parameter values is needed. Non-dimensionalizaton could reduce the number of parameters but this will likely only go so far. Course graining and sensitivity analysis (i.e., lumping together reactions or parameters) would provide an idea of how critical specific reactions are to the behavior. We think a simpler more tractable model is needed.

**RESPONSE:** We respect the Reviewer’s reasonable comments. However, the 85 unknown model parameters are not arbitrarily chosen (see our response to Editor’s 2^nd^ comment above). Our choice for parameter values are extensively justified in S4 Appendix and is also discussed in the Method and Discussion sections. Briefly, values of many of the model parameters are directly adopted (Class 1 parameters) or refined/fine-tuned (Class 2 parameters) from relevant published models in the literature, and the remaining parameter values (Class 3 parameters) are estimated based on scientific reasoning and thermodynamic and kinetic constraints. This approach has enabled us to reproduce the cardinal features (some quantitatively and some qualitatively) of the human mitotic cell cycle function and dysfunction based on recently published experimental data under diverse cellular perturbations. We note here that randomly setting model parameter values should not give rise to a predictable model. Our model is predictive of the cardinal features of the human mitotic cell cycle regulation under normal conditions and its dysregulations under pathological conditions.

We agree with the Reviewer that lumping of interacting reactions or synthesis/degradation processes could have helped in reducing the number of unknown model parameters. However, we wanted to account for all the major biological processes that are experimentally established to have a comprehensive model of the human mitotic cell cycle regulation/dysregulation. It can be seen that the values of certain rate constants and Michaelis-Menten constants have been assigned equal values, as we do not have enough resolution in the available experimental data to identify these parameters. These could be insensitive parameters and/or correlated with each other. We also note here that certain parameter values had to be drastically different to reproduce the cardinal features of the human mitotic cell cycle function/dysfunction (cell cycle oscillation in Figure 2 and its alterations under cellular perturbations in Figures 3-9). To resolve these issues and identify all the unknown model parameters, we need to perform a formal parameter sensitivity analysis. This will also help us to identify if certain interaction reactions or certain synthesis/degradation processes can be lumped in reducing the number of unknown model parameters. Since the current manuscript is already extensive, providing the details of the model development, parameterization, and simulation results focusing on the human mitotic cell cycle biology, we will carry out further analysis of the model in a subsequent manuscript, including model parameter sensitivity analysis and bifurcation analysis.

We also note here that all the state variables in our model are non-dimensional. The protein concentrations are non-dimensionalized with respect to the total Cdk1 concentration. Thus, the number of unknown model parameters cannot be reduced further this way. Another important point is that we have used a model parameterization approach that is used by other established groups in the cell cycle modeling field, e.g. the groups of Drs. John Tyson, Bela Novak, Albert Goldbeter, and others. Model parameters are assigned to qualitatively (if no quantitative data is available) or quantitatively (if quantitative data is available) describe the cell cycle biology in relevant cell system for which the modeling is done. We have further clarified these aspects of our model parameterization approach in the revised manuscript (e.g. **p 13, ln 272-285; p 38, ln 831-841; Supplemental Information S4 Appendix**).

3) A regression fit to known datasets for key protein concentrations kinetics would add more validity to the model and would allow the authors to reduce the model to key state variables that can be fit. Kinetic data and model fits over time, shown on the same figure, is common way to present this and should be shown.

**RESPONSE:** We agree with the Reviewer on this point. Actually, we have done that for which “high-resolution” kinetic data was available. For example, the analysis of the kinetic data in Figure 6B provided us the kinetic parameters governing CDKN1A (p21^CIP1^) with CCNB1:CDK1 (MPF) binding (i.e. the binding constant and Hill coefficient). In many cases, “low-resolution” data was available in the literature, and we have parameterized the model to reproduce the underlying biological phenomena qualitatively rather than quantitatively. This is highly common in the cell cycle modeling field and other modeling field where “high-resolution” quantitative data is not available (see paragraph 3 of our response to comment # 2 above).

4) Experimentally testable predictions of the model should be clearly laid out in a subsection (perhaps in the discussion). This includes the prediction that Kf9 is >> Kr8 and Kf11. This preference of PP2A for specific reactions could be tested easily in vitro by performing kinetic analysis. Although there are no clear predictions made from the model under the various viral stresses simulated (if there are, it would be nice to have them listed together at the end of the discussion section), one could verify the parameter space that induces mitotic collapse in mitotically trapped cells by treating them with increasing doses of the perturbed protein(s) (in Figure 10 simulations) in cell culture. One could also propose decreasing key proteins in a dose dependent manner.

**RESPONSE:** We have further clarified this in the revised manuscript and have also expanded the Discussion section to discuss this (**p 31, ln 671-675**). We have also expanded the Discussion section to list our key findings on the viral dysregulation of the mitotic cell cycle based on our comprehensive model of the human mitotic cell cycle regulation/dysregulation (**p 32-33, ln 698-716**).

5) Typically, the most significant contribution of a model to a field is either (a) explanation of a phenomenon that was unclear or (b) prediction of new experiments that generate non-intuitive outcomes or ideally (c) both. The authors should make clear (in the abstract) in which of these ways the model contributes and then provide background for what was unclear in the field if (a) or (c).

**RESPONSE:** Thank you for your guidance. The purpose of this work was to build an initial base computational model of human mitosis for the purpose of generating new hypotheses and predicting new experiments designed to help understand complex diseases. We have clarified this point in the revised Abstract (**p 2, ln 25-27**). In doing this, we also needed to test the predictability of the model based on what is known experimentally in the literature pertaining to the human mitotic cell cycle regulation/dysregulation. This is reflected in the manuscript abstract, author summary, discussion, and throughout the manuscript where pertinent to mention.

6) Figure 1 is very confusing. Key reactions aren’t displayed; specifically, the MPF phosphorylation of the APC/C complex is left out. The labelling scheme to separate reactions by the order they occur is not visually pleasing or intuitive. A left to right approach with respect to the order of events would be better. One could also separate reactions that occur during a particular stage into boxes and have lines cross the boxes for any transitional interactions.

**RESPONSE:** Thank you for the recommendation. To improve clarity, we have now included simplified wiring diagrams in the new Figure 1. We have updated the text (**p 9, ln 179-181**) and figure legends accordingly **(p 10, ln 202-205)**.

7) The MPF complex is not defined clearly in the text or Figure 1. It is regularly interchanged for CCNB1:CDK1. Consistency should apply, and only one name should be used for the complex after it has been defined as consisting of unphosphorylated CCNB1 and CDK1.

**RESPONSE:** Thank you and apologize for the inconsistency. We have now consistently defined MPF and preMPF throughout the manuscript and in the figures.

8) Would like to see if the same behaviors observed in this model can be seen with a simpler model without so many unknown parameters (i.e., reduce the number of “#” in the Table in the supp info).

**RESPONSE:** It is possible to simulate certain mitotic phenomena observed in this model (e.g. MPF bistable switch through MPF-WEE1-CDC25C interactions; latching-switch mechanism controlling the G2/M transition) through some simpler/reduced models, as described in the Introduction and Discussion of this manuscript. However, these models cannot simulate all the biological phenomena simulated through this comprehensive model. We have extensively justified our rationale for building this comprehensive model in the above responses and throughout the manuscript. One rationale for this collaborative project was to build a more complex model based on changes in relationships between mitotic factors over time using a defined set of experimental conditions. Our approach has resulted in this base quantitative model of low resolution. We will be refining the simulation in future studies using additional published experimental data sets collected using primary human fibroblasts comparing changes in a subset of mitotic factors between infected versus uninfected time points. Human herpesviruses re-configure networks including cell cycle and mitosis to support viral replication. This is also true for transformed cell lines. By building a modular model that captures the salient features and relationships, we hope to both refine and expand the network to help us uncover new hypothesis that otherwise might not be evident using conventional experimental approaches.

**Reviewer #3:**

1) Throughout the manuscript, the authors stated a couple of times that they developed a novel integrated computational model for human mitotic cycle. However, by reading through whole manuscript, I had difficult to find the novelty and the authors did not state clearly what novel aspects are in their study. For example, what new interactions and parameters have included in this model comparing to the many previous models of cell cycle, in particular the mammalian cell cycle models? Are there new bistable switches in the new model in addition to the known ones? What are the new insights gained via current modeling into the experimental observations? I understand that the authors are developing a human model, which could be novel since no human cell cycle model has been developed. But it needs to state clearly what special aspects of model justify it as a human model, not another mammalian cell cycle model, or the novel mechanistic insights that the model provides for the human mitotic cell cycle. I appreciate the efforts that authors had put to develop this complex model, it should be made clear to the readers what is new in the paper.

**RESPONSE:** Thank you for recommendations on how to improve the manuscript. We are grateful to this reviewer and the others for their guidance. We have rewritten the abstract and revised the text throughout the manuscript to more explicitly describe the purpose of the model and its unique features (novelty). At the beginning of the abstract we now state, “The cellular protein-protein interaction network that governs cellular proliferation (cell cycle) is highly complex. Here, we have developed a novel computational model of human mitotic cell cycle, integrating diverse cellular mechanisms, for the purpose of generating new hypotheses and predicting new experiments designed to help understand complex diseases. The pathogenic state investigated is infection by a human herpesvirus. The model starts at mitotic entry initiated by the activities of Cyclin-dependent kinase 1 (CDK1) and Polo-like kinase 1 (PLK1), transitions through Anaphase-promoting complex (APC/C) bound to Cell division cycle protein 20 (CDC20), and ends upon mitotic exit mediated by APC/C bound to CDC20 homolog 1 (CDH1). It includes syntheses and multiple mechanisms of degradations of the mitotic proteins. Prior to this work, no such comprehensive model of the human mitotic cell cycle existed.” (**p 2, ln 24-33**). In addition, we have highlighted unique features (novelty) of the model throughout the document using this or a variation of the phrase, “unique to this model” (e.g. **p 7, ln 149-150; p 8, ln 164-168**). We have revised the Discussion section to succinctly summarize the key features of the model (**p 29, ln 615-620**) and key findings obtained from the model simulations (**p 31, 671-675; p3, ln 711-716**).

2) The first sentence in the Abstract is somewhat awkward by saying that “Our ability to understand … is becoming more difficult due to …” It is becoming more difficult is due to the discovery of more and more new protein interactions and mechanisms. Our ability and the mechanisms are not becoming difficult.

**RESPONSE:** Thank you for identifying the issue. We have completely rewritten the abstract and author summary and have removed the phrase.

3) The abbreviations in the Abstract are not defined.

**RESPONSE:** Thank you for identifying this issue. We have made the correct in the revised Abstract (**p 2**).

4) The authors should state clearly in the Introduction what specific problems they want to solve or the specific rationale for them to develop the human model.

**RESPONSE:** To address the concern, we have revised the Abstract (**p 2, ln 25-28, 40-43**) and the Introduction (**p 7, ln 139-149**) to clearly state the specific problem being studied and the rationale for the problem. Additional comments regarding this point have been provided in response to comment #1.

5) The different arrow types in Fig.1 is very confusing and difficult to digest. For example, protein degradation (e.g., Cyclin B) may occur all the time during the cell cycle, more in one phase while less in another phase. But using solid, open, and dashed arrows to indicate degradation in different phases makes it difficult to read. Another example: APC/C activity peaks at the anaphase, which is indicated by the open arrows, but MPF also peaks at the anaphase, but it is not marked by open arrows.

**RESPONSE:** The rationale for using different arrow styles was to provide a time component to the processes in the wiring diagram. We agree that CCNB1 is degraded throughout the cell cycle duration but to different extent during different phases of the cell cycle. This is represented by degradations using three different processes. Following this and additional comments, we have made modifications to Fig. 1 to more clearly define these processes (**p 10, ln 201-205**) including a description in the inset of the symbols for degradations (**new Fig. 1A**). We have also added two simplified circuit diagrams in new Fig. 1B and C (**p 9, ln 179-181**).

6) Too many lines plotted in a single panel Fig.2, you may plot them in different panels by subgroups. Looks like that Fig.3 is not new but regrouping of Fig.2.

**RESPONSE:** We have removed Fig. 2 and added it into Supplemental Information as noted in the next response.

7) Based on Fig.2, the cycle time is 48 hours. Should be human mitotic cell cycle period around 24 hours?

**RESPONSE:** We have added a statement regarding doubling time variability including the need to adjust this cycle time based on specific cell types (**p 13, ln 278-282**). In the model, cell cycle duration is control by one global scaling parameter and can be easily adjusted to accommodate varying cell types and conditions based on experimental data (e.g. passage number, culture environment, transformed, etc.). We have added the supplemental figure, Fig. S1, which shows both a 48 hr and a 24 hr cycle time in **S5 Appendix**. In future studies, we will be refining the simulation using additional published data sets collected using primary human fibroblasts comparing changes in a subset of mitotic factors between infected versus uninfected time points.

8) I find that it is very difficult to compare the experimental data with the simulation results in Figs. 4, 5, 6. The author should use the same bar graphs as for the experimental data for comparison.

**RESPONSE:** We agree that the data are complex and difficult to compare. The published data sets are from limited time points showing changes in relationships while the model simulations are continuous function of time. The changing relationships over time between mitotic factors are comparable but specific time points in the simulations are not directly comparable to experimental data. Conversion of the simulations to bar graphs at specific times is not feasible. To address the concern, we attempted to convert the bar graphs from published data to line graphs. However, this made interpretations more difficult and we also realized that this approach inaccurately represented the published experimental data, inferring results between points. To address the concern, we have now added arrows accompanying descriptions within the results section showing conservation of changes seen in experimental and simulated data. This is introduced starting with data described for Fig. 3 (**p 14, ln 299-302, 308-309**).

9) Except for some parameters were taken from previous studies, mainly modeling studies, most of the parameters were set arbitrarily and unitless. Can you determine some of the parameters by comparing the simulation results with the experimental data quantitatively? I think that this is one important aspect to justify your model as a human model since the parameters are determined based on human data.

**RESPONSE:** Thank you for this reasonable comment, which coincides with the comments # 2 and 3 of Reviewer 2, which we have addressed above. Briefly, we have used a model parameterization approach that is commonly used in the cell cycle modeling field. Specifically, the model parameters are assigned values to qualitatively (if no quantitative data is available) or quantitatively (if quantitative data is available) describe the cell cycle biology in relevant cell system for which the modeling is done. In our case, since the available experimental data on human mitotic cell cycle regulation/dysregulation is mostly “low-resolution” in nature with which computational model cannot be fitted to estimate the unknown model parameters, we assigned the unknown model parameter values to reproduce the cardinal features of the human mitotic cell cycle regulation under normal conditions. The model is also used to predict mitotic cell cycle dysregulation under different cellular perturbations, including viral infection, for which also similar “low-resolution” experimental data are available in published literature. Our model is clearly predictive of the mitotic cell cycle regulation/dysregulation under diverse cellular conditions. Another note is that we have fitted the model to some available “high-resolution” kinetic data to obtain the kinetic parameters, e.g. see Figure 6B, regarding the kinetics of CDKN1A (p21^CIP1^) binding to CCNB1:CDK1 (MPF), which provided us the estimates of the associated binding constant and Hill coefficient. We have extensively justified our model parameterization approach in the S4 Appendix (further clarified in the revised manuscript), and in the Discussion section.
