# Supplementary material for: Network mechanisms and dysfunction within an integrated computational model of progression through mitosis in the human cell cycle
Source: PLoS Comput Biol. 2020 Apr 6;16(4):e1007733. doi: 10.1371/journal.pcbi.1007733 (PMC7162553; doi:10.1371/journal.pcbi.1007733)
Supplement: Attachment — Submitted filename: Response to Reviewers Comments.docx [file pcbi.1007733.s007.docx]

**Responses to the Reviewer’s Comments:**

**Comment 1.** The title is misleading because the authors have not modeled the complete cell cycle in human cells (no description of progression through G1 and entry into S phase). Rather, they are only describing progression from G2 into mitosis, and then out of mitosis into G1. Their model equations show limit cycle oscillations only because their G1-state morphs into a G2-state without doing DNA replication. This is OK, because the authors only want to describe in detail the molecular changes occurring as a human cell transits into and out of mitosis. But the text should make this clear. And the title should be changed to "... an Integrated Computational Model of Progression through Mitosis in the Human Cell Cycle".

**Response 1:** We thank you for your suggestion. We have changed the title as suggested to better reflect the work undertaken in this manuscript. The revised title is “Network Mechanisms and Dysfunction within an Integrated Computational Model of Progression through Mitosis in the Human Cell Cycle”. We have changed the text to reflect this where necessary.

**Comment 2:** In Fig. 1 the notation 'S1' and 'S2' is a little confusing at first sight. The legend of the figure should say explicitly that S1 is active MPF (CCNB1:CDK1) and S2 is active (phosphorylated) PLK1.

**Response 2:** We are sorry for the confusion. We have further clarified this in the caption for Figure 1 (lines 192-194). We have replaced the text “Signals marked S1 and S2 are medicated by MPF kinase and PLK1 kinase, respectively.” by the text “Here, S1 denotes active MPF kinase (CCNB1:CDK1) and S2 denotes active PLK1 kinase (phosphorylated), which are major signals for many mitotic cell cycle reactions, as indicated.”

**Comment 3:** Regarding 'cell cycle time of 48 h', I think the whole discussion on p 13 is misguided. Because the authors are not simulating the entire human cell cycle (G1-S-G2-M), the period of oscillation of their model equations is unrelated to observed cell division times in human cells. The only relevant parts of their simulations are how the cell enters into M from late G2, and exits from M into early G1. The parameter alpha in their ODEs (Appendix S3) can be adjusted to make this time interval fit the facts in mammalian cells. I don't know what the time period is from late G2 to early G1 in mammalian cells in culture, but I bet it is much shorter than the 50 h in Fig 2.

**Response 3:** We are sorry for the misunderstanding. We completely agree with the reviewer. We have rewritten the text in page 13 to further clarify our points, which is consistent with what the reviewer is alluding to. The revise texts reads as: “We are using a hypothetical 48 hr cycle (oscillation period) to allow for visualization of key changes in the mitotic proteins through the simulations during the cell entering into M phase from late G2 phase and exiting from M phase into early G1 phase. In general, cell doubling times vary by cell type, passage number, and culture conditions. In this model, the mitotic cycle duration (i.e. the duration for the cell to enter into M phase from late G2 phase and exit from M phase into early G1 phase) is controlled by one global scaling factor (α), as described in the Supporting Information, S3 Appendix. Increasing the current scaling factor of 1.4 to 2.8 reduces the mitotic cycle time to 24 hr from 48 hr (Figure S1).”

**Comment 4:** The authors seem to suggest that they can fit their model to any cell cycle duration simply by changing the time-scale factor, alpha. This is true mathematically, but it doesn't make any sense biologically. Cell cycle duration is not adjusted by scaling all rate constants up or down by a constant factor! Rather, cell cycle duration is determined by factors outside the cell-cycle signaling network: by things like nutrient availability, oxygenation, growth factors, cell-cell contacts, etc.

**Response 4:** We completely agree with the reviewer that the cell cycle duration is determined by several biological factors, outside of the cell-cycle signaling network, such as nutrient availability, oxygenation, growth factors, and cell-cell contacts. In our mathematical model, this is controlled by the rate constants of synthesis and degradation of the mitotic proteins and by the forward and reverse rate constants of the interacting reactions in the mitotic biopathway. Correspondingly, these rate constants are implicitly regulated by these biological factors. By proportionally increasing or decreasing the rate constants by a factor **α**, we are able to change the duration of the mitotic cell cycle, as demonstrated in Figure S1 of S5 Appendix. We believe, we are lumping the cumulative effects of all those biological factors into a single parameters **α**. We also think that different rate constants can be differentially regulated by these distinct biological factors, giving rise to alteration in the cell cycle duration. We have added these texts to page 2 bottom and page 3 top of S3 Appendix (lines 39-49). We have also revised middle paragraph of page 2 of S3 Appendix (lines 34-35) to clarify the cell cycle duration that we are referring to be consistent with what we agreed with the reviewer in our response to comment 3 above.
